# Targeting tumor intrinsic TAK1 engages TNF-α-driven cell death through distinct mechanisms and enhances cancer immunotherapy

**DOI:** 10.1038/s41419-025-08013-0

**Published:** 2025-10-16

**Authors:** Jason D. Huska, Kelly J. Doyle, Julie J. Purkal, Cara L. Hrusch, Ryan C. Duggan, Erwin R. Boghaert, Andrew J. Souers, Darren C. Phillips, Stephen K. Tahir

**Affiliations:** Oncology Discovery Research, AbbVie Inc., North Chicago, IL USA

**Keywords:** Targeted therapies, Cell death and immune response

## Abstract

Despite the success of immune checkpoint inhibitors in cancer, many patients do not respond or relapse following treatment. Therefore, new approaches to augment existing immunotherapies are needed. CRISPR screens have revealed the importance of TNF-α signal transduction mediators, such as TAK1, in facilitating tumor susceptibility to cytotoxic T cells. Here, we demonstrate that inhibition of TAK1 in tumor cells lowers the threshold for TNF-α-induced cytotoxicity. Upon TNF-α signaling, pharmacologic inhibition of TAK1 sensitized tumor cells to RIPK1-dependent apoptosis. However, RIPK1-independent apoptosis occurred upon genetic deletion of *Tak1*, suggesting a novel scaffolding function of TAK1 is required to induce RIPK1 kinase activity during cell death. Deleting *Tak1* impaired in vivo tumor growth, enhanced α-PD-1 immunotherapy, and lead to durable anti-tumor memory, dependent on CD8 T cells and intact TNF-α signaling. Our results collectively demonstrate that compromising TAK1 function within tumor cells leverages the cytotoxic capacity of TNF to enhance anti-tumor immunity and generate deeper and more durable anti-tumor immune responses in preclinical models of cancer.

## Introduction

Robust anti-tumor immune responses are essential for the control and clearance of tumors, and therefore, finding new approaches for engaging host immunity against patient tumors is an area of high activity and interest [1]. Immune checkpoint blockade (ICB) molecules such as anti-CTLA4 and anti-PD-1/PD-L1 antibodies have revolutionized the clinical treatment of cancer [2–10]. By relieving the inhibitory actions of their cognate T cell targets, ICB therapies reinvigorate T cells, leading to increased proliferation, cytokine production, and cytotoxic capability, which together, can inflame the tumor microenvironment and increase the durability of patient responses [11–14]. Despite the success of ICB therapies, however, a notable fraction of patients either fail to respond or develop resistance to treatment [15–17]. This highlights the need to develop additional strategies that stimulate the anti-tumor immune response to enhance the utility of cancer immunotherapies.

Several genome-wide CRISPR screens have identified key tumor intrinsic resistance pathways to immunotherapy. These include genes involved in class I MHC regulation and antigen presentation, IFN-γ signal transduction, and PD-L1 expression. The same studies have also identified tumor intrinsic targets that, when genetically depleted, increase the efficacy of immunotherapy [18–23]. One common pathway identified is TNF-receptor (TNFR1)-mediated NF-κB signaling. NF-κB is a prominent transcription factor that is activated by numerous stimuli, including cytokines such as TNF-α. When activated, NF-κB drives transcriptional programs that stimulate both inflammation as well as cell proliferation. Activation of NF-κB also supports cell survival via the expression of anti-apoptotic genes including cIAP1/2, cFLIP, and anti-apoptotic BCL-2 proteins, which together support tumor cell viability [24]. Therefore, manipulating NF-κB activation downstream of TNFR1 may represent a common tumor intrinsic susceptibility to T cell killing.

TNF-α engagement of TNFR1 can initiate both pro-survival and pro-death signaling pathways and its impact on cell fate is determined by several layers of regulation. Upon engagement with soluble TNF-α, TNFR1 induces the rapid formation of a membrane-bound complex (complex I) that activates NF-κB and MAP kinase activation which, in turn, promotes cell survival [25]. Signal transduction within complex I is regulated by a series of polyubiquitin chains generated by cIAP1/2 and LUBAC and conjugated to the kinase RIPK1 [26, 27]. Within complex I, polyubiquitylated RIPK1 recruits and provides binding sites for protein kinases including TAK1 via MAP3K7-binding protein 2 (TAB2) and TAB3 as well as the IκB kinase (IKK) complex. These protein complexes propagate complex I signal transduction by activating downstream kinases such as JUN N-terminal kinase, p38, and NF-κB and culminate in the production of proinflammatory cytokines and pro-survival factors [28]. Defects in the recruitment, ubiquitination, and phosphorylation of proteins in complex I can impair NF-κB activation, and instead promote the formation of a secondary, cytoplasmic complex (complex II) that leads to RIPK1-independent apoptosis via the FADD-dependent recruitment of caspase-8 [29]. Thus, manipulating TNF-α signal transduction represents a potential tumor susceptibility to apoptosis.

A key feature of TNF-α-induced, RIPK1-independent apoptosis is the caspase-8 mediated cleavage of RIPK1 that restrains its kinase activity. However, in certain cellular contexts, kinase-active RIPK1 can promote several modes of death including apoptosis, necroptosis, or pyroptosis [30–34]. These alternative, RIPK1-dependent cell death pathways display hallmarks of inflammatory cell death such as membrane lysis and release of cellular DAMPs, as well as proinflammatory cytokine and chemokine production from dying cells [35–38]. As dying tumor cells can engage and modulate host immune responses, RIPK1-dependent cell death pathways may represent a strategy to enhance host anti-tumor immunity via induction of immunogenic cell death [39–41].

The protein kinase TAK1 has emerged as a key regulator of RIPK1-dependent cell death processes [42]. Recruitment of TAK1 to the TNFR1 limits RIPK1-dependent cell death by driving NF-κB-dependent induction of pro-survival genes as well as phosphorylating MK2, which subsequently phosphorylates RIPK1 to preclude its assembly into complex II [43–45]. Indeed, *TAK1*-deficient cells are hypersensitive to TNF-α-induced cytotoxicity and TNF-α mediated cell death induced by TAK1 inhibition relies on RIPK1 [42, 46]. Upon TNF-α stimulation, TAK1 inhibition can engage both RIPK1-dependent apoptotic and necroptotic machinery depending on the cellular context [46, 47]. Recently, pathogen or pharmacologic blockade of TAK1 has also been shown to activate pyroptosis through a RIPK1:caspase-8 complex that cleaves the pore-forming protein gasdermin D [48–50]. Thus, targeting TAK1 can engage multiple modes of RIPK1-dependent cell death, several of which are proinflammatory and stem from a common stimulus. Interestingly, tumor cells engineered to harbor RIPK1 mutations that prevent polyubiquitination, and thus TAK1 recruitment, displayed enhanced tumor control and synergized with anti-PD-1 immunotherapy [51]. Similarly, activation of RIPK1-dependent apoptosis was found to enhance anti-tumor immunity in models of soft tissue sarcoma [52]. Given that TAK1 activity regulates multiple forms of programmed cell death, we set out to determine if TAK1-regulated, RIPK1-dependent death pathways were present in tumor cells and whether genetic or pharmacological targeting of TAK1 could enhance anti-tumor immune responses.

## Results

### TAK1 inhibition sensitizes tumor cell lines to TNF-α-mediated, RIPK1 kinase-dependent cytotoxicity

**Fig. 1:**
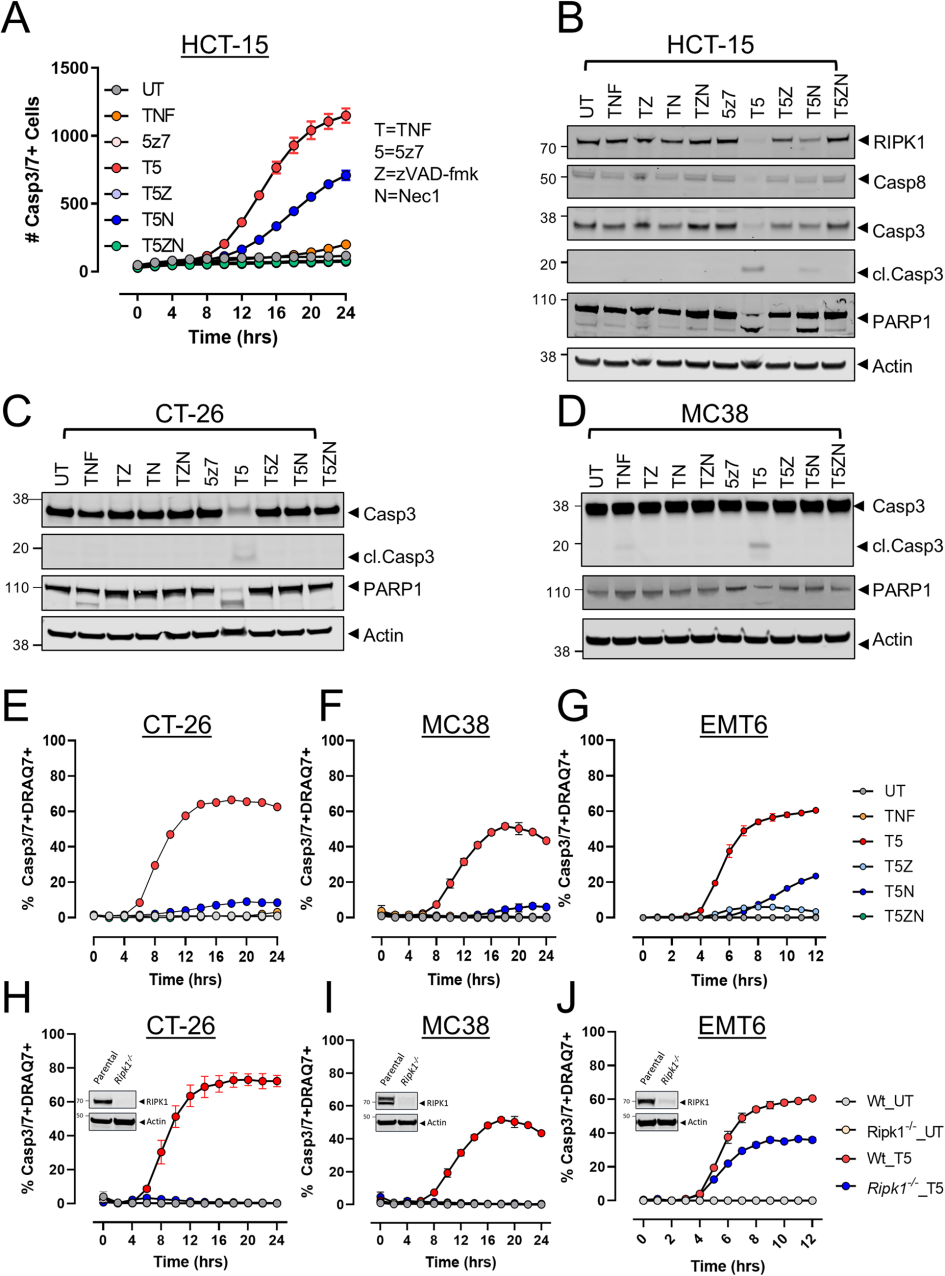
Pharmacologic inhibition of TAK1 sensitizes human and mouse tumor cell lines to TNF-α-mediated, RIPK1-dependent cell death pathways. **A** HCT-15 cells were pretreated with the pan-caspase inhibitor zVAD-fmk (Z, 20 µM, 2 h), the RIPK1 kinase inhibitor necrostatin-1 (N, 30 µM, 1 hr), or both (Z + N) and subsequently treated with TNF-α (T, 12.5 ng/mL) and the TAK1 inhibitor 5z7-oxozeaenol (5z7, 0.125 µM) and the kinetics of caspase-3/-7 activity were assessed. **B** HCT-15 cells were treated as in (**A**) and lysates were probed for the indicated proteins 24 h after TNF-α +/− 5z7 treatment. **C**, **D** Western blot analysis of the indicated proteins in (**C**) CT-26 or (**D**) MC38 mouse tumor cell lines treated as in (**A**). Caspase-3/-7 kinetics were assessed in CT-26 (**E**), MC38 (**F**) and EMT6 (**G**) cells pretreated zVAD-fmk (Z, 20 µM, 2 h), necrostatin-1 (N, 30 µM, 1 hr), or both (Z + N) and subsequently treated with TNF-α (T, 25 ng/mL) and the TAK1 inhibitor 5z7 (5, 0.125 µM). Genetic ablation of *Ripk1* in CT-26 (**H**), MC38 (**I**), or EMT6 (**J**) cell lines rescues viability in response to TNF-α and 5z7. Cells in (**H**–**J**) treated as in (**F**, **G**). Insets depict RIPK1 deletion in the indicated cell line. Data in (**B**–**D**) depict representative results from 2 to 3 independent experiments. Data in (**A**) and (**E**–**J**) represent the mean +/− SD of a representative experiment, *n* = 2–3 independent experiments.

Since RIPK1 activation during cell death is associated with an inflammatory signature and that TAK1 is a key regulator of RIPK1 activity [37, 48, 53], we hypothesized that inhibiting TAK1 in tumor cells could engage TNF-α-mediated, RIPK1-dependent inflammatory cell death. Overnight treatment with TNF-α or the irreversible covalent TAK1 inhibitor 5z7-oxozeaenol (5z7-oxo) had minimal impact on cellular viability and failed to activate caspase-3 in human HCT-15 (Fig. 1A, B) and multiple mouse tumor cell lines (Supplementary Fig. 1). However, the combination of TNF-α and 5z7-oxo induced robust cell killing in all tumor cell lines assessed (Fig. 1A–D, Supplementary Fig. 1A). In human HCT-15 cells, overnight treatment with TNF-α and 5z7-oxo decreased the expression of full length RIPK1 and induced hallmarks of apoptotic cell death including cleavage of caspase-8, activation of caspase-3, and cleavage of PARP1 (Fig. 1B). Pretreatment with the pan-caspase inhibitor zVAD-fmk restored full length RIPK1 and completely blocked PARP1 cleavage, confirming that TNF-α-induced, caspase-dependent apoptosis was driven by TAK1 inhibition (Fig. 1B). Additionally, the expression of RIPK1 and magnitude and kinetics of caspase-3/-7 activation and PARP1 cleavage were also impaired by the RIPK1 inhibitor necrostatin-1, confirming activation of RIPK1-dependent apoptosis in HCT-15 cells treated with TNF-α and 5z7-oxo (Fig. 1A, B). Similarly, TNF-α and TAK1 inhibition also induced caspase-3 activation and PARP1 cleavage in multiple mouse tumor cell lines and could be rescued by either caspase or RIPK1 inhibition, indicative of RIPK1-dependent apoptosis induction (Fig. 1C–G, Supplementary Fig. 1B). In agreement with these findings, deletion of *Ripk1* in CT-26, MC38, and EMT6 mouse tumor cells inhibited caspase-3 activation and cell death induced by TNF-α and 5z7-oxo (Fig. 1H–J). Together, these data indicate that TAK1 inhibition sensitizes human and mouse tumor cell lines to TNF-α-mediated cytotoxicity and that RIPK1-dependent apoptosis is active in tumor cell lines of multiple origins.

### Genetic ablation of *Tak1* in tumor cells drives TNF-α-dependent cytotoxicity

**Fig. 2:**
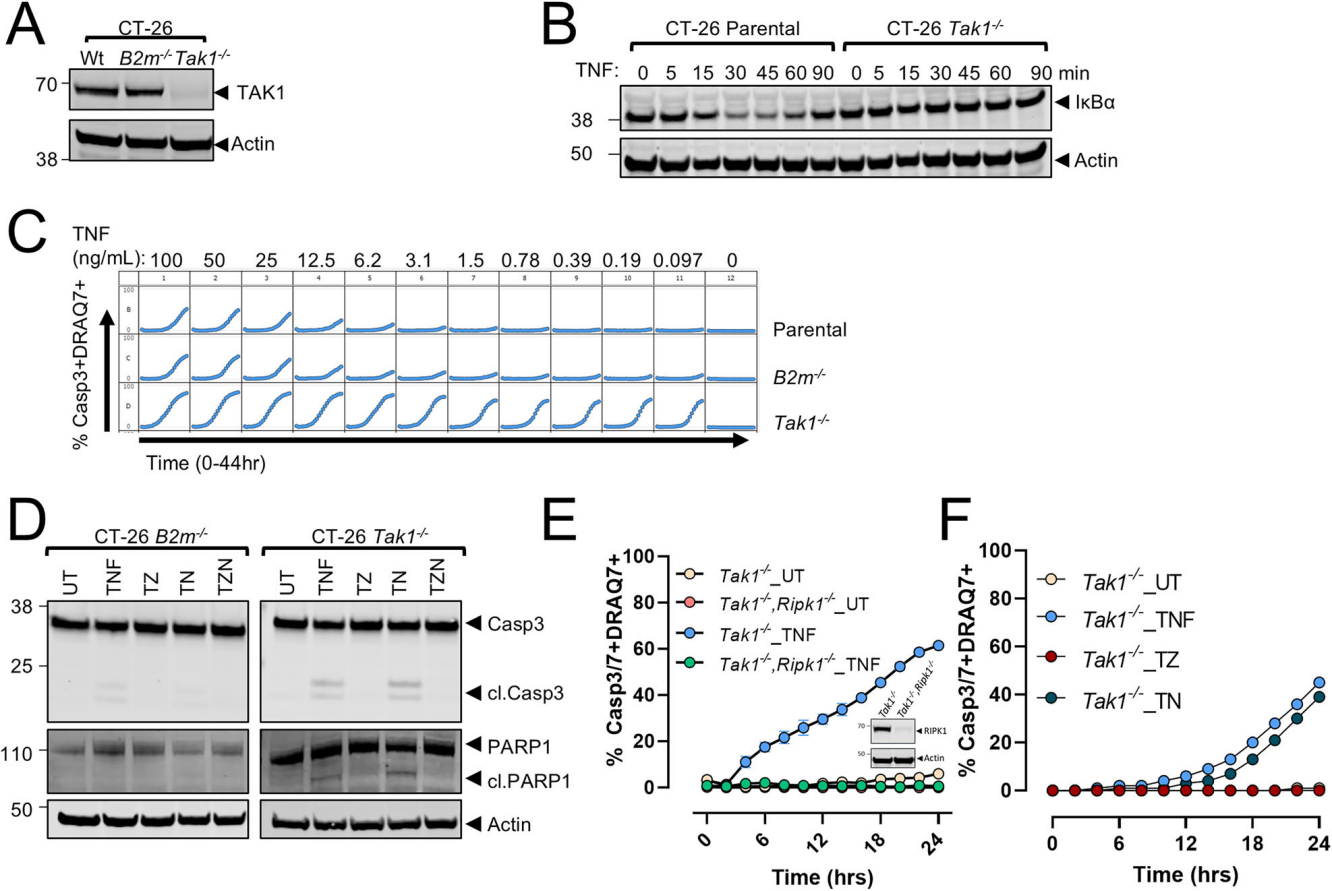
Genetic inhibition of *Tak1* increases TNF-α cytotoxicity but compromises RIPK1 kinase-dependent apoptosis. **A** CRISPR/Cas9 RNP mediated deletion of *Tak1* in mouse CT-26 cells. Β2-microglobulin (*B2m)* deletion was performed in parallel and served as a CRISPR/Cas9 deletion control. **B** Parental and *Tak1*^*−/−*^ CT-26 cells were treated with 25 ng/mL of TNF-α and degradation of IκBα was assessed at the indicated time. **C** *Tak1*^*−/−*^ CT-26 cells were treated with the indicated concentration of TNF-α and assessed for capsase-3/-7 and DRAQ7 activity over the duration of 44 h, capturing images every 2 h. **D** *Tak1*^*−/−*^ *or* CRISPR control (*B2m*^*−/−*^) CT-26 cells were pretreated with zVAD-fmk (Z, 20 μM), necrostatin-1 (N, 30 μM), or both and subsequently treated overnight with TNF-α (25 ng/mL). Lysates were probed for the indicated proteins. **E** *Tak1*^*−/−*^*, Tak1*^*−/−*^*/Ripk1*^*−/−*^ CT-26 were treated with 25 ng/mL TNF-α and caspase-3/-7 activity and membrane permeability was assessed every 2 h for 24 h. Inset depicts RIPK1 knockout efficiency. **F** *Tak1*^*−/−*^ CT-26 cells were pretreated for 2 h with zVAD-fmk (Z, 20 μM) or necrostatin-1 (N, 30 μM) and subsequently treated with TNF-α (T, 25 ng/mL). Caspase-3/-7 activity and membrane permeability were assessed every 2 h for 24 h. Data in (**A**, **B**, **D**) depict representative results from 2 to 3 independent experiments. Data in (**C**, **E**, **F**) depict the mean +/− SD of a representative experiment, *n* = 3 independent experiments.

To confirm that TAK1 inhibition increases tumor cell sensitivity to TNF-α cytotoxicity, the gene encoding TAK1 (*Map3k7*, referred to here as *TAK1* [human] or *Tak1* [mouse]) was deleted in multiple tumor cell lines using CRISPR/Cas9 RNP technology. Deleting *Tak1* in CT-26, EMT6, MC38, or HCT-15 (*TAK1*) cells did not compromise in vitro cell growth compared to parental or CRISPR control cells (*B2m*^*−/−*^), indicating that TAK1 is dispensable for normal proliferation (Fig. 2A, Supplementary Figs. 2A, D, I, 3A). These data also indicate that unlike bone marrow derived macrophages, TAK1 deletion does not result in spontaneous TNF production in tumor cells [54, 55]. When stimulated with TNF-α, parental CT-26 cells rapidly degraded IκBα, a key inhibitor of NF-κB (Fig. 2B), demonstrating an intact TNF-α-responsive NF-κB signaling pathway. Conversely, when exposed to TNF-α, *Tak1*^*−/−*^ CT-26 cells failed to degrade IκBα (Fig. 2B), and instead exhibited considerable cytotoxicity (Fig. 2C). Similar results were obtained for *Tak1*^*-/-*^ EMT6 and MC38 cells (Supplementary Fig. 2E, F, J, K), as well as human *TAK1*^*−/−*^ HCT-15 cells (Supplementary Fig. 3B, C). Cell death in *Tak1*^*−/−*^ tumor cells was characterized by activation of caspase-3 and PARP1 cleavage, together indicating TNF-α-induced apoptosis (Fig. 2D). As expected, deleting *Ripk1* in *Tak1*-deficient CT-26 cells rescued the TNF-α-induced death, consistent with reports of TNF-α-mediated, RIPK1-dependent apoptosis induction upon TAK1 depletion or inhibition (Fig. 2E) [46–48]. Surprisingly, and in contrast to parental CT-26 cells treated with TNF-α and 5z7-oxo, necrostatin-1 failed to rescue TNF-α-induced apoptosis in *Tak1*-deficient CT-26 cells (Fig. 2F, Supplementary Fig. 2B, C). This indicates that in the absence of TAK1 protein expression, CT-26 tumor cells instead underwent RIPK1 scaffold-dependent, yet kinase-independent, apoptosis in response to TNF-α. Similarly, RIPK1-independent apoptosis induction occurred in *Tak1*-deficient EMT6, MC38, and *TAK1*^*−/−*^ HCT-15 cells lines treated with TNF-α, yet RIPK1 kinase-dependent apoptosis occurred using various pharmacological TAK1 inhibitors (Supplementary Figs. 2G, H, L, M, 3D, E, 4A, B). These data indicate the pharmacological inhibition of TAK1 does not phenocopy genetic ablation of *Tak1* and suggests that structural elements of TAK1 are critical for TNF-α-mediated, RIPK1 kinase-dependent apoptosis.

### Caspase-8 mediated cleavage of RIPK1 limits RIPK1-dependent apoptosis in *Tak1*-deficient tumor cells

**Fig. 3:**
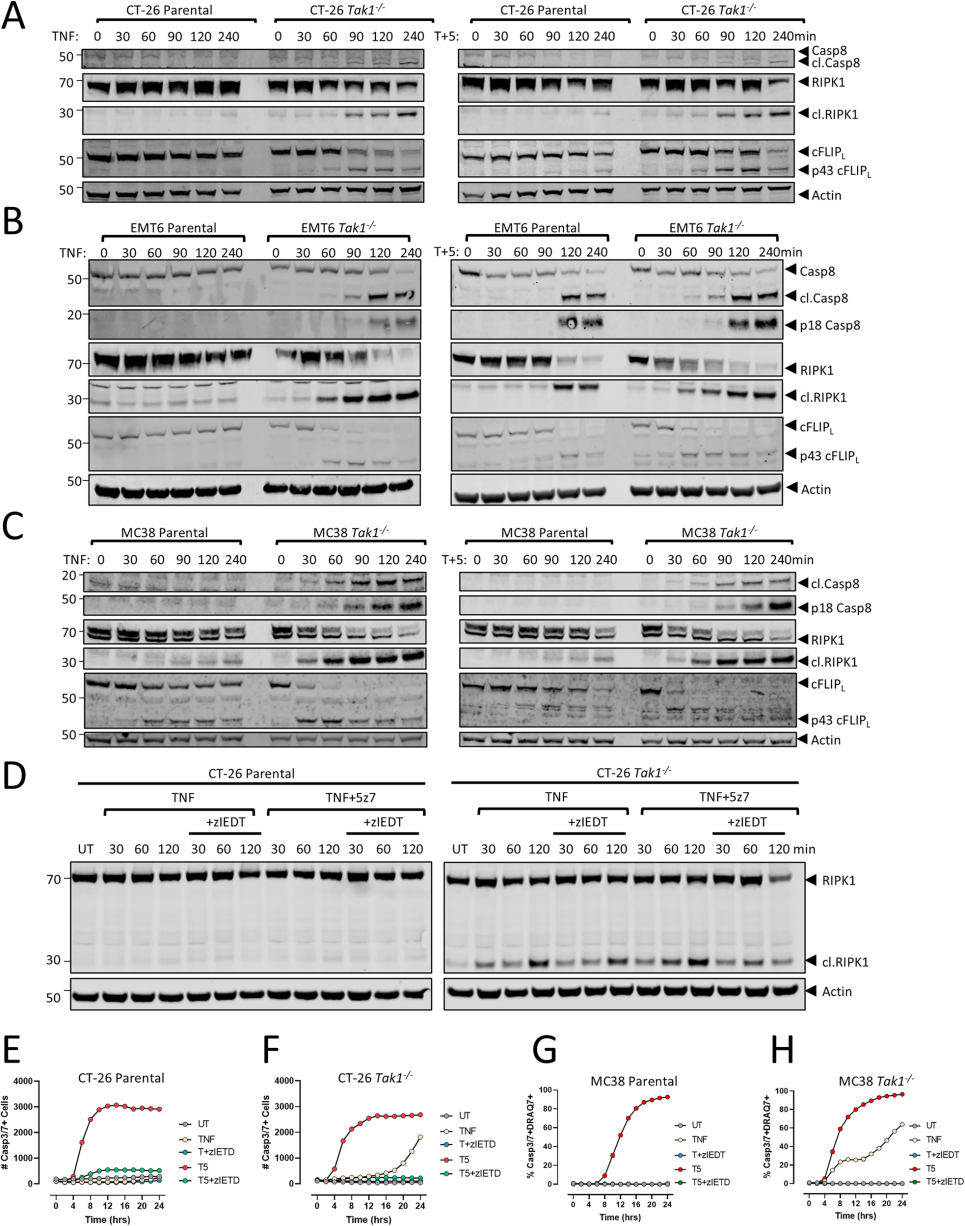
TNF-α signaling induces caspase-8 dependent RIPK1 cleavage in *Tak1*^*−/−*^ cells. CT-26 (**A**), EMT6 (**B**), or MC38 (**C**) parental or *Tak1*^*−/−*^ were treated with 25 ng/mL TNF-α -/+ 5z7-oxo (0.125 μM T + 5) and lysates were assessed for cleavage of the indicated proteins at the specified times. **D** Parental or *Tak1*^*−/−*^ CT-26 cells were pretreated with the caspase-8 inhibitor zIEDT-fmk (zIEDT, 20 μM, 2 h) and subsequently treated with TNF-α (25 ng/mL) in the presence or absence of 5z7-oxo (5z7, 0.125 μM). Cleavage of RIPK1 was assessed at the indicated times. **E**, **F** The number of caspase-3/-7+ cells were assessed in parental (**E**) or *Tak1*^*−/−*^ (**F**) CT-26 cells pretreated with the caspase-8 inhibitor zIEDT-fmk (zIEDT, 40 μM, 2 h) and subsequently treated with TNF-α (25 ng/mL) in the presence or absence of 5z7-oxo (5z7, 0.125 μM). Images were captured every 2 h for 24 h. **G**, **H** Parental (**G**) or *Tak1*^*−/−*^ MC38 (**H**) cells were treated as in (**E**, **F**) and caspase-3/-7 activity and membrane permeabilization was assessed every 2 h for 24 h. Data in (**A**–**D**) representative results from 2-3 independent experiments. Data in (**E**–**H**) are the mean +/− SD of duplicate measurements, *n* = 3 independent experiments.

The observation that *Tak1* deficient cancer cells underwent RIPK1 kinase-independent apoptosis in response to TNF-α was unexpected given the previous reports of RIPK1-dependent apoptosis induction using other models of TAK1 inhibition [46–48]. A hallmark of RIPK1-independent apoptosis is the cleavage of RIPK1, limiting its ability to function as a kinase. We therefore assessed RIPK1 protein expression in *Tak1*^*−/−*^ cancer cells stimulated with TNF-α to understand if RIPK1 is cleaved in the absence of TAK1 protein expression. In *Tak1*^*−/−*^ CT-26 cells treated with TNF-α with or without 5z7-oxo, RIPK1 was readily cleaved within 60 min of stimulation (Fig. 3A). Similar and more pronounced results were observed in *Tak1*^*−/−*^ EMT6 and MC38 cells treated with TNF-α −/+ 5z7-oxo, with full length RIPK1 exhibiting almost complete cleavage by 240 min after treatment (Fig. 3B, C). In all cases, similarly treated parental cells maintained full length RIPK1, even at 240 min after treatment, a timepoint just prior to caspase-3 activation (Figs. 1F and 3A). Therefore, in tumor cells with intact TAK1 protein expression, RIPK1 is maintained in its full-length form where its kinase domain likely becomes activated during TNF-α-mediated, TAK1 inhibitor-induced apoptosis. Previous studies have demonstrated that TAK1-mediated NF-κB activation induces the expression of cFLIP_L_ to inhibit TNF-α-mediated apoptosis and prevent RIPK1 kinase-dependent death [56], inferring that cFLIP_L_ expression may be reduced in *Tak1-*deficent cells. However, in *Tak1*^−/−^ CT-26, EMT6, and MC38 cells, basal levels of cFLIP_L_ were unchanged compared to parental controls (Fig. 3A–C). Moreover, cFLIP_L_ was also readily cleaved in *Tak1*-deficient tumor cells with similar kinetics as RIPK1 upon TNF-α treatment (Fig. 3A–C). Therefore, cFLIP_L_ expression does not alter RIPK1 cleavage in *Tak1*-deficient tumor cells.

Both RIPK1 and cFLIP_L_ are canonical capase-8 substrates [57, 58]. Following TNFR1 engagement, the kinase activity of RIPK1 is restrained by caspase-8 mediated cleavage leading to RIPK1 kinase-independent apoptosis [36, 59, 60]. Therefore, we hypothesized that in TNF-α treated *Tak1*-deficient cancer cells, caspase-8 mediated cleavage of RIPK1 may limit its kinase activity during apoptosis. Supporting this hypothesis, pretreatment with the caspase-8 specific inhibitor zIETD-FMK partially reduced RIPK1 cleavage in TNF-α treated, *Tak1*^*−/−*^ CT-26 cells and completely restored viability of both *Tak1*-deficient CT-26 and MC38 cells treated with TNF-α or their parental counterparts treated with TNF-α + 5z7-oxo (Fig. 3D–H). This highlights the importance of caspase-8 initiating TNF-α-driven apoptosis induced by TAK1 inhibition. In agreement with reports that RIPK1-mediated cell death retains aspects of immunogenicity [40], dying parental CT-26 tumor cells maintained full length RIPK1 (Fig. 3A, E), exhibited NF-κB pathway activation and released multiple proinflammatory cytokines and chemokines in response to TNF-α and 5z7-oxo (Supplementary Fig. 5A–D). Cytokine and chemokine production in *Tak1*^*−/−*^ CT-26 cells were completely inhibited under similar conditions, even though this treatment induced similar complex I formation and robust apoptosis. Therefore, the absence of RIPK1-dependent cell death in *Tak1*-deficient cells limits cytokine production, likely due to a failure to maintain full length RIPK1 during the death process.

### RIPK1-dependent apoptosis in cancer cells requires TAK1 structural elements

**Fig. 4:**
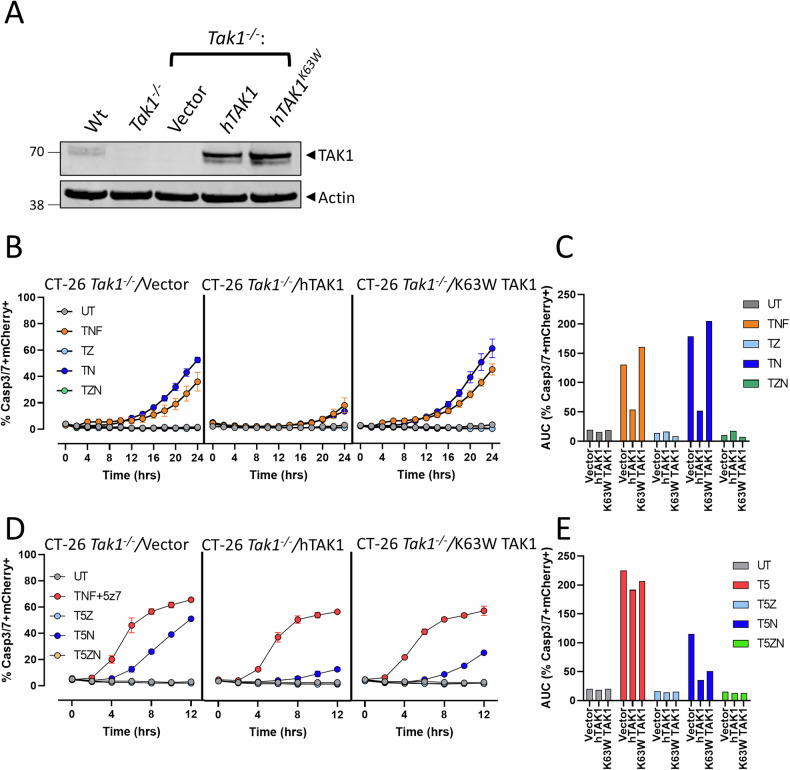
TAK1 reconstitution rescues TNF-α -mediated cytotoxicity and restores RIPK1-dependent cell death. **A** Expression of human TAK1 protein in *Tak1*^*−/−*^ CT-26 cells transduced with empty vector or lentiviruses expressing either wild-type or kinase dead (K63W) TAK1 compared to the parental cell lines. **B** TAK1 reconstituted cells were pretreated with zVAD-fmk (Z, 20 µM, 2 h), necrostatin-1 (N, 30 µM, 1 h), or both (Z + N) and subsequently treated with TNF-α (T, 25 ng/mL). Caspase-3/-7 activity was assessed every 2 h for 24 hr. **C** Area under the curve (AUC) analysis for each caspase-3/-7 curve depicted in (**B**). **D** TAK1 reconstituted cells were treated as in (**B**), with the addition of the TAK1 inhibitor 5z7-oxozeaenol (5, 0.125 μM) during TNF-α treatment. Caspase-3/-7 activity was assessed every 2 h for 12 h, where maximum caspase-3/-7 activity was observed. **E** AUC analysis for the caspase-3/-7 curves depicted in (**D**). Data in (**B**) and (**D**) depicts the mean +/− SD of a representative experiment, *n* = 4 independent experiments. AUC plots in (**C**) and (**E**) are representative of the given experiment in panels (**B**) and (**D**), respectively.

The discrepancy between the ability to engage TNF-α-mediated RIPK1 kinase-dependent cell death with an inhibitor of TAK1, but not by genetic deletion, suggests structural features of TAK1, in addition to enzyme inhibition, may be required to activate RIPK1. Previous studies have employed a Cre/Loxp based method when generating *Tak1* deficient cells. Using this approach, the ATP-binding domain of *Tak1* is removed via Cre-mediated recombination. However the remainder of the gene is spliced back in frame [61]. This results in a truncated TAK1 protein lacking kinase function that we speculate can be recruited to the TNFR1 and act as a dominant negative, kinase-dead scaffold during induction of RIPK1-dependent cell death. Therefore, to assess if TAK1 acts as a molecular scaffold to engage RIPK1-dependent apoptosis, *Tak1*^*−/−*^ CT-26 cells were reconstituted with either wild-type or kinase dead (K63W) human TAK1 (hTAK1) (Fig. 4A). Reconstitution with hTAK1 efficiently rescued TNF-α-mediated cell death, while empty vector or K63W TAK1 reconstituted cells retained caspase-3/-7 activity following TNF-α stimulation (Fig. 4B, C). As expected, all reconstituted cells displayed robust caspase-3/-7 activation and cell death when treated with TNF-α + 5z7-oxo (Fig. 4D, E). However, while necrostatin-1 failed to rescue empty vector reconstituted cells, reconstitution with wild-type or K63W TAK1 restored the ability of necrostatin-1 to rescue cell death (Fig. 4D, E). Collectively these data indicate that hTAK1 can also act as a scaffold to engage TNF-α-induced, RIPK1 kinase-dependent apoptosis.

### *Tak1* deficiency sensitizes tumor cells to CD8 T cell-derived, TNF-α-driven cytotoxicity

**Fig. 5:**
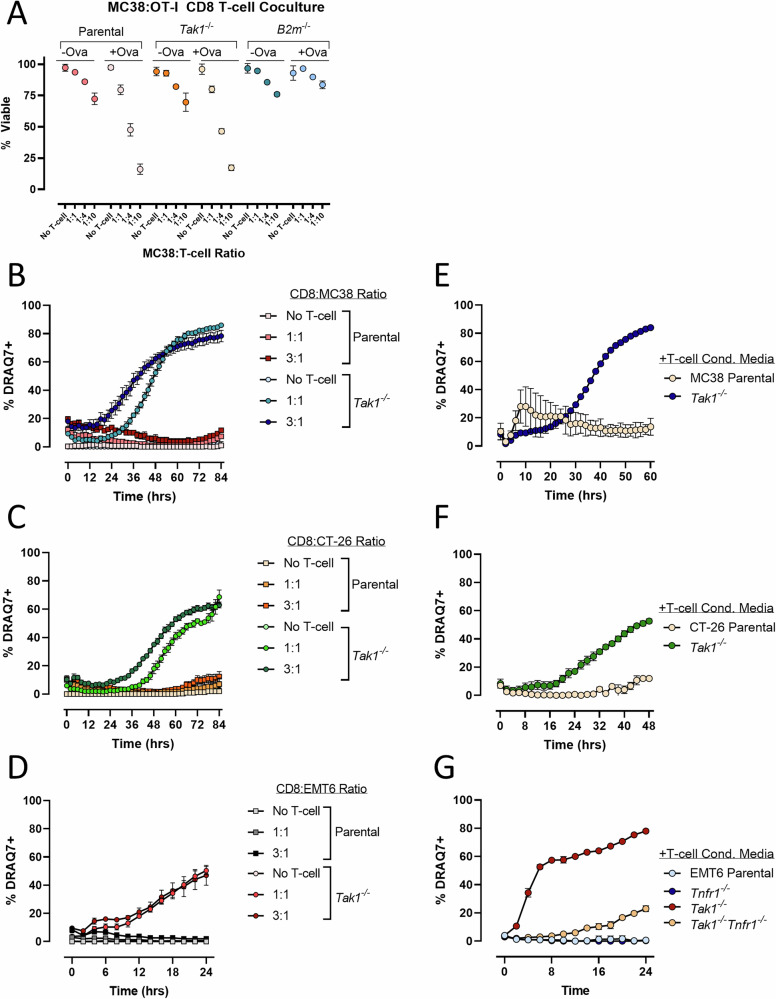
*Tak1*-deficient tumor cells are susceptible to CD8 T cell derived, TNF-α-driven cytotoxicity. **A** MC38 cells lacking *Tak1* or *B2m* expression were pulsed with the MHC-I restricted chicken ovalbumin SIINFEKL peptide and subsequently cultured with increasing ratios of OT-I CD8 T cells, previously activated for 48 h by CD3 and CD28 stimulation. Tumor cell viability was assessed 24 h later by flow cytometry, using CD8 surface staining to discriminate T cells from tumor cells. **B**–**D** CD8 T cells isolated from naïve mouse spleens (Balb/c for CT-26 and EMT6, C57BL/6 for MC38) were activated by CD3 and CD28 stimulation in vitro and subsequently cultured with (**B**) MC38, (**C**) CT-26, or (**D**) EMT6 tumor cells lacking *Tak1* expression. Viability was assessed using DRAQ7 uptake, imaging cells every 2 h for the indicated times. **E**–**G** *Tak1-*deficient MC38 (**E**) or CT-26 (**F**) cells were cultured in media derived from 48 h activated CD-8 T cells. Viability was assessed via DRAQ7 uptake. **G** Conditioned media from 48 h activated CD8 T cells was added to EMT6 cells deficient for *Tak1*, *Tnfr1*, or *Tak1* and *Tnfr1* and viability was assessed via DRAQ7 uptake. All panels represent the mean +/− SD from a single experiment, *n* = 2 independent experiments.

Our in vitro evidence indicates that in the absence of *Tak1* expression, tumor cells become increasingly sensitive to TNF-α-induced apoptosis (Fig. 2C). Because TNF-α can be produced by activated T cells [23], we assessed if *Tak1*^−/−^ tumor cells would exhibit increased cell death when co-cultured with activated CD8 T cells. Parental or *Tak1*^*−/−*^ MC38 cells were pulsed with the MHC-I restricted chicken ovalbumin (Ova) SIINFEKL peptide and co-cultured overnight with increasing ratios of activated OT-I CD8 T cells. OT-I CD8 T cells express a transgenic TCR that recognizes the SIINFEKL peptide presented within the binding cleft of class I MHC molecules and therefore, results in peptide-MHC-dependent killing. Interestingly, in the presence of Ova peptide and activated OT-I CD8 T cells, *Tak1*^*−/−*^ MC38 cells exhibited similar sensitivity to antigen-dependent CD8 T cell killing as their parental counterparts (Fig. 5A). Deleting *B2m*, the structural component of MHC-I, abolished CD8 T cell killing as expected, confirming the specificity of the interaction. These data suggest that tumor cells lacking *Tak1* are equally susceptible to direct TCR:MHC mediated killing mechanisms. Interestingly, when co-cultured with activated CD8 T cells isolated from the spleens of wild-type naïve mice, *Tak1*^*−/−*^ MC38 cells but not parental MC38 cells exhibited substantial cell death (Fig. 5B). Similar results were obtained when *Tak1*^*−/−*^ CT-26 or EMT6 cells were co-cultured with activated, wild-type CD8 T cells (Fig. 5C, D) and indicates that *Tak1* deficiency sensitizes tumor cells to an antigen-independent CD8 T cell killing mechanism. To assess if soluble factors (i.e. cytokines) from activated CD8 T cells were responsible for mediating the killing of *Tak1*^*−/−*^ tumor cells, we cultured MC38, CT-26, and EMT6 cells lacking *Tak1* in conditioned media from CD8 T cells activated by CD3/CD28 stimulation. The CD8 T cell conditioned media induced robust killing in all three *Tak1*^*−/−*^ tumor cell lines (Fig. 5E–G). Interestingly, the magnitude and kinetics of cell death induced by the conditioned media correlated with their respective sensitivity to TNF-α-induced killing (EMT6 > CT-26 > MC38, Fig. 2C and Supplementary Fig. 2F, K). Co-deletion of the TNF Receptor 1 (*Tnfrsf1a*, referred to here as *Tnfr1*) in *Tak1*^*−/−*^ EMT6 cells largely prevented cell death induced by the CD8 T cell conditioned media (Fig. 5G), supporting a role for CD8 T cell derived TNF-α in mediating *Tak1*^*−/−*^ tumor cell killing. Together, these data indicate that TAK1 expression in tumor cells protects from TNF-α-induced cytotoxicity mediated by activated CD8 T cells.

### T cell-independent in vivo tumor control in *Tak1*-deficient EMT6 tumor model

**Fig. 6:**
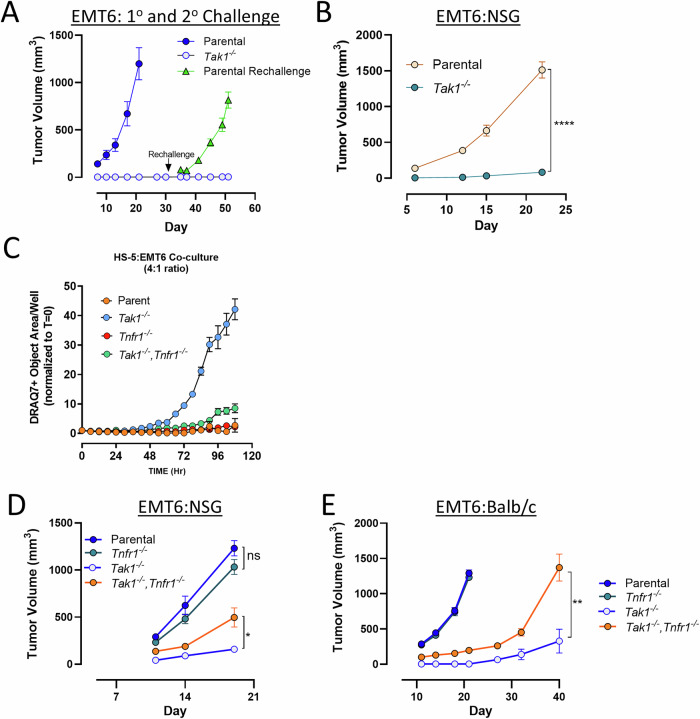
*Tak1* deficiency limits tumorigenesis independent of adaptive immunity in a TNF-α hypersensitive tumor model. **A** Immunocompetent mice (BALB/c, *n* = 30/group) were implanted with pooled clones from *Tak1*-deficient or parental EMT6 tumor cells and tumor growth was monitored over time. All *Tak1*-deficient EMT6 tumors (30/30) were rejected by Day 7 while parental tumors exhibited robust growth. After 4 weeks of undetectable growth, mice that previously rejected *Tak1*-deficient EMT6 tumors were subsequently inoculated on the opposing flank with parental EMT6 cells and tumor growth was monitored. Tumor growth on the original flank was also monitored to assess if the primary *Tak1*-deficient tumors would form. **B** Parental or *Tak1*-deficient EMT cells were implanted into the flanks of immunodeficient mice (NSG, *n* = 10/group) and tumor growth was monitored over time. **C** Parental, *Tak1*^*−/−*^*, Tnfr1*^*−/−*^, or *Tak1*^*−/−*^*/Tnfr1*^*−/−*^ EMT6 cells were co-cultured at a 4:1 ratio with human HS-5 stromal cells and tumor cell death was tracked over time via DRAQ7 uptake. **D**, **E** EMT6 parental, *Tnfr1*^*−/−*^*, Tak1*^*−/−*^, or *Tak1*^*−/−*^*/Tnfr1*^*−/−*^cells were implanted into the flanks of immunodeficient NSG (**D**, *n* = 10/group) or immunocompetent Balb/c mice (**E**, *n* = 10/group) and tumor growth was monitored. Tumor growth curves represent the mean tumor volume +/− SEM. Statistical differences between tumor volumes at the final measurement was determined using a two-way ANOVA with Tukey’s multiple correction where *p < 0.05; **p < 0.01; and ****p < 0.0001 was considered significant.

Because *Tak1*-deficient tumor cells displayed increased sensitivity to in vitro CD8 T cell killing, we speculated that this would translate to more robust in vivo tumor control. Interestingly, *Tak1*-deficient EMT6 tumor cells failed to establish when implanted into immunocompetent mice, with 100% of animals exhibiting complete tumor clearance by day 7 (Fig. 6A). Because tumor control was so rapid, it seemed unlikely that this effect was mediated by CD8 T cells or other adaptive immune mechanisms. We hypothesized that if T cells were activated during the primary tumor response, then anti-tumor memory T cells should control a secondary tumor challenge with parental EMT6 cells. Therefore, mice that had originally rejected a *Tak1*^*−/−*^ EMT6 tumor were subsequently rechallenged with parental EMT6 cells and tumor growth was monitored. Parental EMT6 tumors efficiently grew in all animals that had previously cleared *Tak1*^*−/−*^ tumors, suggesting anti-tumor memory T cell responses failed to generate in these mice (Fig. 6A). Surprisingly, and in support of an adaptive immunity-independent control mechanism, *Tak1*^*−/−*^ EMT6 tumors also failed to establish in immunodeficient NSG mice (Fig. 6B). These data indicate that in vivo control of *Tak1*^*−/−*^ EMT6 tumors does not involve T cells.

TNF-α is a pleotropic cytokine that can be produced by numerous cell types, including non-immune cells such as fibroblasts [62]. To determine if non-immune cell sources of TNF-α could mediate cell death in tumor cells lacking *Tak1*, we co-cultured EMT6 *Tak1*^*−/−*^ cells with HS-5 stromal cells. HS-5 cells are derived from human fibroblasts and can produce TNF-α during normal growth [63]. Because HS-5 is a human cell, we first confirmed that human TNF-α was capable of inducing cell death in *Tak1*-deficient EMT6 cells (data not shown). HS-5 co-culture had little impact on the viability of parental EMT6 cells. However, *Tak1*^*−/−*^ EMT6 cells exhibited robust cell death in the presence of HS-5 fibroblasts (Fig. 6C). Cytotoxicity appeared to be mediated predominantly by TNF-α, as viability was largely restored in EMT6 *Tak1*^*−/−*^*,Tnfr1*^*−/−*^ double knockout cells (Fig. 6C). Therefore, stromal cell derived TNF-α can mediate in vitro killing of *Tak1*-deficient EMT6 tumor cells.

To address if non-lymphoid cell derived TNF-α exerted in vivo anti-tumor effects on *Tak1* EMT6 cells, we implanted EMT6 *Tak1*^*−/−*^*/Tnfr1*^*−/−*^ double knockout cells into the flanks of immunodeficient NSG mice. As before, *Tak1*^*−/−*^ EMT6 cells failed to grow in NSG mice whereas robust tumor growth was observed upon inoculation with both parental and *Tnfr1*^*−/−*^ EMT6 cells (Fig. 6D). However, codeletion of *Tnfr1* from *Tak1*^*−/−*^ EMT6 cells partially restored their ability to form tumors in the absence of adaptive immune pressure, supporting a role for non-lymphoid derived TNF-α limiting tumor growth in the absence of TAK1 function. (Fig. 6D). Similarly, EMT6 *Tak1*^*−/−*^*/Tnfr1*^*−/−*^ double knockout cells also formed tumors in immunocompetent mice (Fig. 6E). However, in both immune backgrounds, EMT6 *Tak1*^*−/−*^*/Tnfr1*^*−/−*^ tumors exhibited slower growth kinetics than parental or *Tnfr1*-deficient EMT6 cells (Fig. 6D, E) indicating that additional host pressures or tumor intrinsic deficits mediated by loss of *Tak1* function can also decrease in vivo tumor cell fitness.

### *Tak1* deficiency limits in vivo tumorigenesis, enhances immune checkpoint therapy and induces anti-tumor memory responses

**Fig. 7:**
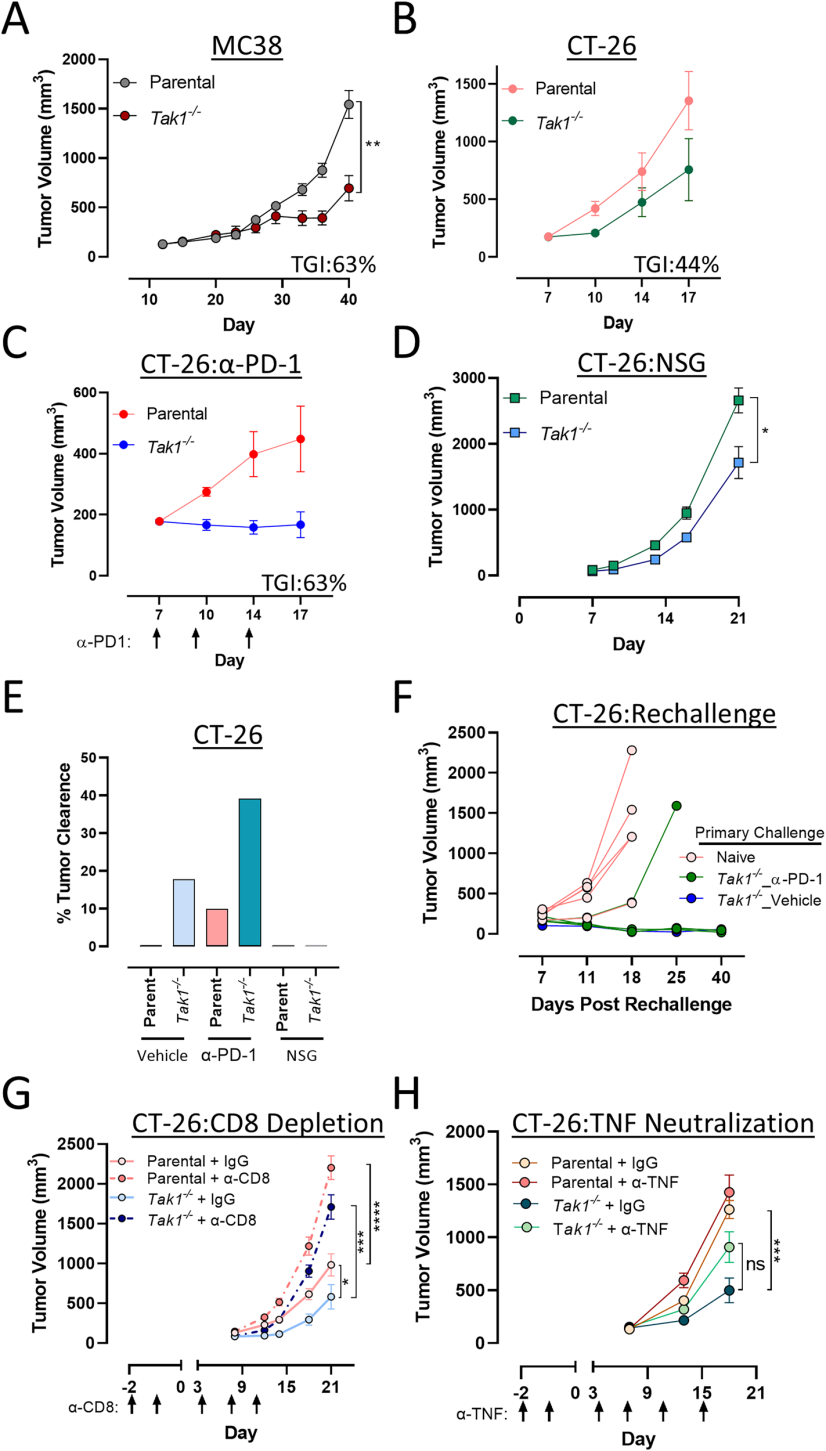
*Tak1* deficiency limits in vivo tumorigenesis, enhances α-PD-1 checkpoint therapy, and induces anti-tumor memory responses. **A** Immunocompetent mice (C57BL/6, *n* = 15/group) were implanted with pooled clones from *Tak1*-deficient or parental MC38 cells and tumor growth was monitored. **B**, **C** Immunocompetent mice (BALB/c, *n* = 8/group) were implanted with *Tak1*-deficient CT-26 cells (**B**) and treated at the indicated days with an α-PD-1 antibody (**C**, 10 mg/kg) and tumor growth was monitored. **D** Immunodeficient mice (NSG, *n* = 10/group) were implanted with *Tak1*-deficient CT-26 cells and tumor growth was monitored. **E** Quantification of the percentage of complete tumor clearance from panels (**B**–**D**). **F** Balb/c mice implanted with *Tak1*-deficient CT-26 tumors and exhibiting complete responses were rechallenged with CT-26 parental cells and tumor growth was monitored. Naïve mice challenged with parental CT-26 cells served as controls. **G** CT-26 parental and *Tak1*-deficient tumor growth was monitored in Balb/c mice administered an α-CD8 depleting antibody for two sequential days prior to tumor engraftment and during tumor progression at the indicated days (*n* = 10/group). **H** CT-26 parental and *Tak1*-deficient tumor growth was assessed in Balb/c mice administered an α-TNF-α neutralizing antibody (15 mg/kg) at the indicated days (*n* = 10/group). Tumor growth curves represent the mean tumor volume +/− SEM. Statistical differences between tumor volumes at the final measurement was determined using a two-way ANOVA with Tukey’s multiple correction where *p < 0.05; **p < 0.01; and ****p < 0.0001 was considered significant.

We next assessed how *Tak1* deletion impacted the in vivo tumorigenesis of MC38 and CT-26 tumor cells, both of which exhibited less in vitro TNF-α responsiveness than the EMT6 model. When implanted in immunocompetent mice, *Tak1*-deficient MC38 tumors demonstrated slower growth compared to parental MC38 tumors (Fig. 7A, 63% tumor growth inhibition). Similarly, *Tak1*^*−/−*^ CT-26 cells also exhibited impaired tumorigenesis in immunocompetent mice (Fig. 7B, 44% tumor growth inhibition), with several animals showing complete tumor clearance (5/28, Fig. 7E). When combined with α-PD-1 immunotherapy, growth of *Tak1*-deficient CT-26 tumors was further reduced and the percentage of mice exhibiting complete tumor clearance increased (Fig. 7C, E, 63% tumor growth inhibition, 11/28 cleared tumors). Therefore, unlike the EMT6 tumor model described above, activation of anti-tumor T cell immunity enhances control of *Tak1*^*−/−*^ tumor cells that exhibit moderate in vitro TNF-α sensitivity. Supporting this, regression of *Tak1*^*−/−*^ CT-26 tumors was dependent on adaptive immunity as immunodeficient mice failed to clear *Tak1*^*−/−*^ CT-26 tumors (Fig. 7D, E). The ability to control *Tak1*-deficient CT-26 tumors also conferred protection to a secondary rechallenge with parental CT-26, indicative of a robust anti-tumor memory response (Fig. 7F).

Control of CT-26 tumors completely relied on CD8 T cells, as parental and CT-26 *Tak1*^*−/−*^ tumor growth was indistinguishable in mice treated with an α-CD8 depleting antibody (Fig. 7G). Although CD8 T cells were required for *Tak1*^*−/−*^ CT-26 tumor control, we did not observe an increase in their intratumoral frequency or activation, even with α-PD-1 immunotherapy (Supplementary Fig. 6A–L). We therefore hypothesized that TNF-α production from CD8 T cells rather than their overall number within the tumor was more important for *Tak1*^*−/−*^ tumor control in this model. To test this, we treated mice inoculated with parental or *Tak1* deficient CT-26 cells with an α-TNF-α neutralizing antibody and assessed tumor growth. Although TNF-α neutralization had no impact on the growth of parental CT-26 tumors, treatment with an α-TNF-α neutralizing antibody partially restored growth of *Tak1*^*−/−*^ CT-26 tumors (Fig. 7H). Collectively, our data indicates that TAK1 functions to protect tumor cells from cytotoxic TNF-α signaling and an anti-tumor CD8 T cell response. Impaired TAK1 activity in tumor cells lowers the threshold to TNF-α driven cytotoxicity, which can enhance the anti-tumorigenic activity of current checkpoint immunotherapeutics.

## Discussion

The ability to engage anti-tumor host immunity is a key determinant of the clinical efficacy of cancer immunotherapeutics. As such, strategies that enhance immune cell activation or lower the threshold for tumor killing by immune cells have the potential to provide more durable responses in cancer patients. Here, we investigated TAK1-mediated, TNFR1 signal transduction as a means to manipulate tumor cell sensitivity to TNF-α-driven cytotoxicity. In multiple tumor cell lines, genetic loss of TAK1 function prevented TNF-α-induced activation of NF-κB, which would otherwise signal towards cell survival. Instead, impeding TAK1 during TNF-α signaling shunts tumor cells towards cell death pathways. This results in robust in vivo anti-tumor immunity where *Tak1* deficient tumor cells displayed significantly lower growth compared to their parental counterparts.

Our studies demonstrate that control of *Tak1*-deficient tumors was primarily due to T cell mediated killing in a TNF-α-dependent manner. Although *Tak1*-deficient CT-26 cells displayed impaired growth in immunodeficient mice, the presence of adaptive immune pressure significantly increased tumor control, with several animals displaying no measurable tumors. The addition of α-PD-1 immunotherapy further enhanced control of *Tak1*^*−/−*^ CT-26 tumors, extended survival, and increased the incidence of tumor free mice, further supporting a role for host immunity in limiting *Tak1*^*−/−*^ tumor growth. Either the depletion of CD8 T cells or the administration of an α-TNF-α-neutralizing antibody enabled growth of *Tak1*^*−/−*^ CT-26 tumors in vivo. Similarly, *Tak1*^*−/−*^ EMT6 tumor growth in immunocompetent mice could be restored by co-deletion of the TNF receptor. Interestingly, control of *Tak1*^*−/−*^ EMT6 tumor growth was also observed in immunodeficient mice and could be restored by co-deletion of the TNF receptor. This indicates that TNF-α from non-T cell sources, including stromal cells within the tumor microenvironment, can potentially contribute to limit tumor growth when targeting tumor intrinsic TAK1. This finding also represents a distinct advantage in targeting tumor intrinsic TAK1, as it increases the breadth of cells that can contribute to the overall anti-tumor response beyond cytotoxic T cells.

TAK1 plays a central role in coordinating the outcome of TNFR1 signaling both by initiating NF-κB and MAP kinase signaling cascades, and also by restraining the cytotoxic potential of RIPK1 [43, 45, 64, 65]. We therefore hypothesized that TAK1 inhibition in tumor cells might enhance anti-tumor immune responses via induction of RIPK1-dependent immunogenic cell death pathways. While we were able to demonstrate a cell death-promoting role for the RIPK1 scaffold in both paternal and *Tak1*^*−/−*^ tumor cells treated with TNF-α/5z7-oxo or TNF-α alone, respectively, a noteworthy difference was the requirement for RIPK1 kinase activity. While the TNF-α dependent cell death induced by pharmacological inhibition of TAK1 relied on RIPK1 kinase activity, cell death in tumor cells deficient in *Tak1*^*−/−*^ did not. These data indicate that in the absence of TAK1, tumor cells undergo RIPK1 kinase-independent cell death instead, and are reminiscent of the RIPK1-independent cell death reported in *Tak1*-deficient mouse bone marrow-derived macrophages following TLR stimulation [36, 37]. As the scaffold of RIPK1 is also implicated in driving NF-κB mediated gene expression, we assessed if maintaining full length RIPK1 after pharmacological inhibition of TAK1 could maintain inflammatory signaling during the cell death cascade. In agreement with previous studies demonstrating the immunogenic potential of kinase-active RIPK1 [39, 40], cell death in parental cells treated with TAK1 inhibitors coincided with NF-κB pathway activation and the release of proinflammatory cytokines and chemokines, both of which were absent in TNF-α-treated, *Tak1*^*−/−*^ tumor cells. Thus, although both the scaffold and kinase function of RIPK1 are required for to drive TNF-mediated tumor cell death in the absence of TAK1 activity, TAK1 is also necessary to limit RIPK1 cleavage, allowing sustained NF-κB activation. Although tumor growth is impaired by *Tak1* deletion, it is interesting to speculate that restoring RIPK1-kinase dependent apoptosis in *Tak1*-deficient tumors may further enhance the anti-tumor response by maintaining proinflammatory cytokine production from the dying tumor cells.

It is currently unclear why RIPK1 is so readily cleaved in *Tak1*-deficient cells treated with TNF-α. Several studies have pointed to a role for cFLIP in limiting caspase-8 activity during RIPK1-dependent cell death pathways [66]. However, cFLIP levels did not appear to be altered in our cell lines lacking *Tak1*. Moreover, cFLIP was also cleaved with similar kinetics as RIPK1 in TNF-α-treated *Tak1*-deficient cells. Other studies have implicated upstream complex I components in controlling caspase-8 and RIPK1 activity upon TAK1 inhibition [67, 68]. However, the expression of caspase-8, TRADD, RIPK1, and FADD did not correlate with RIPK1 kinase-dependent cell death in the mouse tumor cell lines employed and we noted that RIPK1 was equally ubiquitylated within complex I of both parental and *Tak1*^*−/−*^ CT-26 cells, suggesting the TNF-α signaling pathway components upstream of TAK1 were intact and functional (Supplementary Fig. 5C–E). Instead, we speculate that TAK1 may act as a molecular scaffold, required to limit caspase-8 activity and drive RIPK1 activation. In agreement, reconstitution of *Tak1*^*−/−*^ cells with an enzymatically inactive TAK1 (K63W) was able to restore the RIPK1 kinase-dependent apoptotic death following treatment with TNF-α and 5z7-oxo. At first glance, our results appear to differ from published reports that have shown RIPK1 kinase activation in *Tak1*-deficient cells [46, 54, 69]. However, this discrepancy may stem from the different approaches utilized. In multiple studies a Cre/Loxp based approach is used to generate *Tak1* deficiency where, following Cre-mediated DNA recombination that removes the ATP-binding site of TAK1, the remainder of the gene is spliced back in frame [61]. This results in a truncated TAK1 protein lacking kinase function that, while expressed at a reduced level, could conceivably be recruited to the TNFR1 and act as a dominant negative, kinase dead protein. Our approach to generating *Tak1*-deficient tumor cell generated a complete knockout of TAK1, eliminating all protein expression. Therefore, our studies highlight a new scaffolding role of TAK1 required to activate TNF-α-mediated RIPK1 kinase-dependent apoptosis. While future studies will explore TAK1 scaffolding function in further detail, we cannot exclude the possibility that TAK1 may also have a direct function in limiting caspase-8 activity or RIPK1 activation via direct phosphorylation [64].

Taken together, our study provides evidence that manipulating the TNFR1 signaling pathway via TAK1 inhibition provides a benefit to anti-tumor control mechanisms by shifting the response away from survival mediated by NF-κB activation and instead towards immunogenic cell death. These results support the rational for targeting tumor-intrinsic TAK1 as a means of harnessing anti-tumor host immunity and enhancing the efficacy of immune checkpoint therapeutics.

## Methods

### Cell lines

HCT-15, C1498, EL4, EMT6, LL/2, TGP52, and TGP61 cell lines were obtained from ATCC. The MC38 cell line was obtained from the National Cancer Institute (NIH, Rockville, MD). The CT-26 cell lines were obtained from ATCC or MD Anderson (I. J. Fidler laboratory). The RENCA cell line was obtained from MD Anderson and the AE17 cell line was obtained from Sigma. HCT-15, AE17, CT-26, and RENCA cell lines were cultured in RPMI-1640 supplemented with 10% FBS, 2 mM Glutamax, and antibiotic/antimycotic solution. C1498, EL4, EMT6, LL/2, MC38, TGP52, and TGP61 lines were maintained in DMEM supplemented with 10% FBS, 2 mM Glutamax, and antibiotic/antimycotic solution. All cell lines were verified by STR analysis and routinely confirmed to be free from mycoplasma infection.

### Reagents

Recombinant human TNF-α and recombinant murine TNF-α, and IL-7 were from Peprotech (Cranbury, NJ). Recombinant murine Flag-tagged TNF-α was from Enzo (Farmingdale, NY). 5z7-oxozeaenol and Takinib were from Cayman Chemical (Ann Arbor, MI). HS-276 was from MedChemExpress. zVAD-FMK, zIETD-FMK, and recombinant murine IL-2 and IFN-γ were from R&D Systems (Minneapolis, WI). Necrostatin-1 and ABC-FP was synthesized by AbbVie Inc.

### Generation of knockout cell lines

Genomic deletion was performed using the ALT-R CRISPR-Cas9 system from IDT (Coralville, IA). Briefly, equimolar amounts of two different CRISPR RNA (crRNA) guides (Table 1) and the ATTO 550-labled tracrRNA were combined and incubated at 37 °C for 30 min to form the RNA duplex. The duplex was combined with Alt-R S.p. Cas9 Nuclease V3 (IDT) and the resulting RNP complex was subsequently introduced into 0.5–1 × 10^6^ cells of the indicated cell line by electroporation using a NEON Transfection System (Thermo Fisher Waltham, MA). The following settings were used: CT-26, MC38, and EMT6 (3 × 10 ms pulse, 1600 V) or HCT-15 (2 × 40 ms pulse, 1130 V). At 24 h after electroporation, the ATTO 550+ population was sorted, expanded, and assessed for target protein expression. When protein levels in the edited population were reduced >90%, the total population was used for experiments. When protein reduction was <90%, the ATTO 550 population was single cell sorted into wells of a 96-well plate, clones were expanded and assessed for protein expression via western blot (TAK1, RIPK1) or flow cytometry (β2m or TNFR1). For MC38 and EMT6 in vivo tumorigenesis experiments, 8–12 parental or *Tak1*^*−/−*^ clones were combined to form a pseudo-population and then implanted into mice.

**Table 1:** crRNA sequences used in this study.

Murine *Tak1* crRNA1: 5’-TCGAAGTTCAGGACCTGCGA
Murine *Tak1* crRNA2: 5’-GTTGTCGGAAGAGGAGCTTT
Murine *Ripk1* crRNA1: 5’-TGTGAAAGTCACGATCAACG
Murine *Ripk1* crRNA2: 5’-AGTCGAGTGGTGAAGCTACT
Murine *Tnfr1* crRNA1: 5’- CGGACAGTCACTCACCAAGT
Murine *Tnfr1* crRNA2: 5’- ACTAGTCCAGTGACCCCTGA
Murine *B2m* crRNA1: 5’-CACGCCACCCACCGGAGAAT
Murine *B2m* crRNA2: 5’-CTGGTGCTTGTCCACTGAC
Human *TAK1* crRNA1: 5’-CATCTCACCGGCCGAAGACG
Human *TAK1* crRNA2: 5’-GATCGACTACAAGGAGATCG
Human *B2M* crRNA1: 5’-CGTGAGTAAACCTGAATCTT
Human *B2M* crRNA2: 5’-AAGTCAACTTCAATGTCGGA

### TAK1 lentiviral reconstitution

Viruses expressing wild-type or kinase-dead TAK1 (K63W) were generated by GeneWiz (South Plainfield, NJ) using the pLVX-EF1-apha-IRES-mCherry vector (Takara Bio, San Jose, CA). *Tak1*^*−/−*^ CT-26 cells were transfected with lentiviruses using Lenitblast Premium (Takara Bio), and 72 h later were sorted by mCherry fluorescence, taking the bottom 20% of the expressing population.

### In vitro cell death assessment

Cells were plated in 96- or 384-well plates, allowed to attached overnight, and treated with the indicated doses of TNF-α and 5z7-oxozeaenol. Viability was measured the next day using Cell Titer Glo following the manufacturer’s specifications (Promega, Madison, WI). For determining cell death pathways, cells were first pretreated with zVAD-FMK (20 μM), necrostatin-1 (30 μM), or zIETD-FMK (40 μM) for 2 h at 37 °C. During the pretreatment, the caspase-3/-7 fluorogenic sensor (1 μM, Sartorius, Ann Arbor, MI) and Draq7 (1.5 μM, BioLegend, San Diego, CA) or Cytotox Red (5 μM, Sartorius) were added. Following pretreatment, cells were treated with the indicated compounds and confluency, caspase-3/-7 activity, and membrane permeability were tracked every 1–2 h using an IncuCyte S3 system (Essen Biosciences, Ann Arbor, MI). The resulting images were used to define dead cells as apoptotic (Casp3/7+Draq7+) or non-apoptotic (casp3/7-Draq7+).

### CD8 T cell isolation and in vitro coculture

CD8 T cells were isolated from the spleens of either OT-I or wild type C57BL/6 mice using negative selection beads according to the manufacturer’s instructions (Miltenyi Bioscience, Gaithersburg, MD) and cultured for 48 h with plate-bound α-CD3 (1 μg/mL, 145-2C11, BioLegend) and soluble CD28 (5 μg/mL, 37.51, BioLegend) in media containing IL-2 (5 ng/mL), IL-7 (5 ng/mL), and IL-12 (10 ng/mL). For coculture experiments, tumor cells were seeded into wells of a 96-well plate the day before the experiment (CT-26 at 1 × 10^4^ cells/well, MC38 at 2 × 10^4^ cells/well, EMT6 at 0.5 × 10^4^ cells/well). Activated CD8 T cells were added to the tumor cells at the indicated ratios and cells were cultured overnight in an IncuCyte S3, imaging every 2 h. For OT-I coculture, MC38 cells were first pulsed for 2 h with Ova SIINFEKL peptide (10 ng/mL, Sigma, St. Louis, MO), washed, and activated CD8 T cells were added. The next day, cells were stained with an α-CD8 antibody to distinguish T cells from tumor cells. For experiments using T cell conditioned media, the tumor cell growth media was replaced with media from 48 h activated CD8 T cells. As a control, tumor cell growth media was replaced with standard T cell growth media.

### HS-5 coculture

HS-5 cells expressing GFP were generated by transducing cells with Nuclight Green lentivirus (puro) (Sartorius, Bohemia, NY) following the manufacturer’s instructions. Cells were seeded into 96 well plates at a 4:1 ratio with EMT6 cells of the indicated genotypes. Draq7 (1.5 μM) was added to each well and cell death was monitored using IncuCyte S3, imaging every 2 h.

### Immunoblotting

Cells were collected and lysed in ice cold RIPA buffer containing HALT protease and phosphatase inhibitors (Thermo Scientific) and benzonase (Millipore Sigma, Kankakee, IL). Lysates were rotated at 4 °C for 30 min and subsequently clarified by centrifugation at 20 × *g* for 15 min. The soluble fraction was removed, and protein concentration was determined using the using the Pierce bicinchoninic acid assay (Thermo Scientific). Thirty micrograms of protein lysate were size separated through 4–12% SDS-PAGE gels (Invitrogen, Carlsbad, CA) and transferred to PVDF membranes (Invitrogen) using the iBlot 2 semi-dry transfer system. Membranes were blocked in Intercept PBS protein-free blocking buffer (Li-Cor, Lincoln, NE) for one hour and incubated overnight with the indicated primary antibodies. Membranes were washed, incubated with the IRDye secondary antibodies (LI-COR, 926-68020 and 926-32213), and imaged with the Odyssey CLx (LI-COR). Primary antibodies from Cell Signaling Technologies (Danvers, MA): caspase-3 (9662, 1:1000), caspase-8 (4927, 1:1000), cleaved caspase-8 (8592, 1:1000), cFLIP (56343, 1:1000), IκBα (4812, 9242, 1:1000), PARP (9532, 1:1000), RIPK1 (3493, 1:1000), and TAK1 (4505, 1:1000); from Abcam (Boston, MA): RIPK1 (ab178420, 1:2000) and TAK1 (ab109526, 1:1000); from BD Pharmingen (San Diego, CA): PARP1 (51-6639GR, 1:1000); from Enzo: caspase-8 (ALX-804-242-C100, 1:1000); and from MP Biomedicals (Irvine, CA): actin (691002, 1:10,000).

### Complex I immunoprecipitation

CT-26 cells (10 × 10^6^ in 10 mL cell culture media) were seeded into 150 cm^2^ culture plates and allowed to adhere overnight. The next day, cells were treated with 1 μg/mL FLAG-TNF-α for the indicated times and subsequently lysed on ice in 1 mL of Pierce IP lysis buffer (Thermo Scientific). Cell lysates were cleared by centrifugation and subjected to overnight immunoprecipitation using a FLAG-M2 affinity gel matrix according to manufacturer’s specifications (Millipore Sigma). The next day, beads were washed in IP lysis buffer and immunoprecipitated proteins were eluted by boiling in 2X SDS sample buffer. Immunoprecipitants were loaded into SDS-PAGE gels and subjected to immunoblotting described above.

### In vitro cytokine assessment

Cell culture supernatant was collected from overnight treated cells and cytokine production was assessed using the proteome profiler mouse cytokine array kit, panel A following the manufacturer’s specifications (R&D Systems).

### Mice

All protocols were approved by the Institutional Animal Care and Uses Committee (IACUC) at AbbVie Inc. and performed in accordance with National Institutes of Health Guide for the Care and Use of Laboratory Animals. All animals were housed under standard laboratory conditions with *ad libitum* access to food and water in a temperature and humidity-controlled room with a 12-h light:dark cycle. All experiments were performed during the daytime (between 9:00 AM and 3:00 PM). Adult female BALB/c, C57BL/6 mice (20 grams, Charles River Laboratories, Wilmington, MA, USA), OT-I, and NSG mice (25 g, Jackson Laboratories, Bar Harbor, ME) were housed in groups of ten in solid bottom Plexiglas cages equipped with constant air circulation in an Association for Assessment and Accreditation of Laboratory Animal Care International (AAALAC) approved facility. Animals were acclimated to the laboratory environment for 5–7 days before the study.

### In vivo tumorigenesis and rechallenge studies

Mice were inoculated with 1 ×10^5^ parental or *Tak1*^*−/−*^ (*Map3k7*) CT-26, EMT6, or MC38 cells at 0.1 mL/ mouse in a mixture of 1:1 cell media and Matrigel (BD Biosciences, Bedford, MA) via subcutaneous right flank injection. Effect size and sample size calculations were previously determined using a power calculation set to 80%, whereby 8–10 animals per group are required to achieve statistical significance of 0.05 when tumor reduction of a test article is 45–48% compared to vehicle controls measured at the same time. Animals were randomly distributed into treatments groups (*n* = 10/group) when tumors reached mean tumor volume of ~150 mm^3^. Tumor volumes were determined unblinded using a caliper ellipsoid model: L × W^2^/2, where the larger (L) and smaller (W) perpendicular dimensions are measured. Tumor measurements were regularly performed one to three times per week. Mean group body weights were taken at least once per week, coinciding with tumor measurements. Where indicated, mice were treated with anti-mouse PD-1 antibody synthesized at AbbVie (17D2[mu IgG2a/k] DANA) 10 mg/kg, Q4Dx3, IP). For the CD8 depletion protocol, mice were injected with anti-mouse CD8 (BioXCell; BP0061, New Haven, CT) or IgG2a Isotype control (BioXCell; BP 00085) at 10 mg/kg i.p. at the dose schedule of Day -2, Day -1 prior to flank inoculation of cells, then again on Days 4, 8, and 11 post inoculation. Animals participating in the anti-TNF-α trial received injections of Abbvie synthesized anti-mu-TNF-α 8C11-1E10 (TNF-α mAb) or PBS at 15 mg/kg i.p. at the dose schedule of Day-2, Day -1 prior to flank inoculation of cells, then again on Days 4, 8, 11 and 15 post inoculation Complete response was predefined as three consecutive measurements of <25 mm^3^. Animals that displayed complete response were subsequently rechallenged with parental tumor cells, using the method described above.

Percent tumor growth inhibition (TGI) was calculated at the end of study using the following formula: %TGI (tumor growth inhibition) = 1 – (mean tumor volume of treatment group/ mean tumor volume of treatment control group) × 100. Tumor growth delay (TGD) was determined as the percentage increase of the median time period for the treatment group to reach an arbitrary tumor volume of 1000 mm^3^ relative to the vehicle control group and was calculated using the following formula: % TGD (tumor growth delay) = (T − C)/C × 100, where T is the median time to endpoint of treatment group and C is the median time to endpoint of treatment control group. All studies had a predefined endpoint of 1000 mm^3^ and not to exceed 3000 mm^3^. When an individual tumor volume reached a size ≤20% of the weight or the mouse or just exceeded the predefined maximum limit, as determined by micro caliper measurement, the individual mouse was euthanized by over-exposure to inhalation anesthesia or CO_2_.

### Tumor digestion and immunophenotyping

Animals were euthanized via Isoflurane^®^ (Baxter) and flank tumors were excised and placed into a gentleMACS ™ C tube (Miltenyi Biotec) filled with 2.5 ml of tumor dissociation buffer. Tumors were dissociated using the mouse tumor dissociation kit following the manufacturer’s protocol (Miltenyi Biotec). Briefly, whole tumors were dissociated and incubated in enzyme mix for 40 min at 37 °C on a GentleMACS Octo Dissociator. The cell suspension was passed through 70-μm strainers (Falcon/Corning) and washed with an equal volume of PBS. The resulting single-cell suspension was counted and up to 2 ×10^6^ live cells were stained with the indicated antibodies (Table 2). Tumor infiltrating immune cells were analyzed by flow cytometry using an Aurora Spectral Analyzer (Cytek Biosciences, Fremont, CA).

**Table 2:** Flow cytometry antibodies used in this study.

Antigen	Color/Format	Clone	Company	Catalog
CD278 (ICOS)	BUV395	C398.4A	BD Biosciences	565884
Viability	LIVE/DEAD Fixable Blue	N/A	Thermo Fisher Scientific	L23105
CD69	BUV737	H1.2F3	BD Biosciences	612793
CD8a	BUV805	53-6.7	BD Biosciences	612898
FoxP3	eFluor 450	FJK-16s	Thermo Fisher Scientific	48-5773-82
CD44	BV 510	IM7	BioLegend	103044
PD-1	BV 605	29F.1A12	BioLegend	135220
Ki-67	BV 650	B56	BD Biosciences	563757
CD62L	BV 785	MEL-14	BioLegend	104440
CD45	Alexa Fluor 532	30-F11	Thermo Fisher Scientific	58-0451-82
Granzyme B	PerCP-Cy5.5	QA16A02	BioLegend	372212
CTLA-4	PE-CF594	UC10-4F10-11	BD Biosciences	564332
CD3	Spark NIR 685	17A2	BioLegend	100262
CD25	APC-R700	PC61	BD Biosciences	565134
CD49b	APC-Cy7	DX5	BioLegend	108920
CD4	APC-Fire 810	GK1.5	BioLegend	100479
β2 microglobulin	APC	A16041A	BioLegend	154506
CD120a (TNFR1/p55)	APC	55R-286	BioLegend	113006

### Bioinformatic and statistical analyses

Unless otherwise specified, GraphPad Prism was used for statistical analyses and noted in figure legends. For tumor growth inhibition, comparisons between groups were conducted on the final day of measurement, after any group median reached a predefined endpoint of 1000 mm^3^. Comparisons between groups were conducted using a two-way ANOVA with Tukey’s multiple correction where *p < 0.05; **p < 0.01; ***p < 0.001; and ****p < 0.0001. Gene expression analysis was performed on curated data accessed from Gene Expression Omnibus using accession numbers GSE247046 and GSE217132. All data were normally distributed, assessed using the Shapiro–Wilk normality test using GraphPad Prism. All data was assumed to have equal variance.

## Supplementary information

Supplementary Information

Uncropped westerns

## Data Availability

The datasets generated and/or analyzed during the current study are available from the corresponding authors on reasonable request.
