# Supplementary material for: Targeting tumor intrinsic TAK1 engages TNF-α-driven cell death through distinct mechanisms and enhances cancer immunotherapy
Source: Cell Death Dis. 2025 Oct 16;16(1):725. doi: 10.1038/s41419-025-08013-0 (PMC12532812; doi:10.1038/s41419-025-08013-0)
Supplement: Supplementary file 1 — Supplementary Information [file 41419_2025_8013_MOESM1_ESM.pdf]

Supplementary Information

Targeting Tumor Intrinsic TAK1 Engages TNF-α-Driven Cell Death Through Distinct Mechanisms and Enhances Cancer Immunotherapy

Jason D. Huska[1], Kelly J. Doyle[1], Julie J. Purkal[1], Cara L. Hrusch[1], Ryan C. Duggan[1], Erwin R. Boghaert[1], Andrew J. Souers[1], Darren C. Phillips[1] and Stephen K. Tahir[1*]

[1]Oncology Discovery Research, AbbVie Inc., 1 North Waukegan Rd., North Chicago, IL 60064

*Correspondence to: Stephen Tahir, E-mail: stephen.k.tahir@abbvie.com

Supplementary Materials

This supplementary file includes seven figures S1 to S7.

Supplementary Figure 1. The TAK1 inhibitor 5z7-oxozeaenol sensitizes mouse tumor cell lines to RIPK1 kinase-dependent, TNF-α-mediated cytotoxicity.

Supplementary Figure 2. Characterization of *Tak1*-deficient murine tumor cell lines.

Supplementary Figure 3. Characterization of *TAK1*-deficient HCT-15 cells.

Supplementary Figure 4 Differential requirement for RIPK1 kinase activity during TNF-mediated cell death in tumor cells upon TAK inhibition vs genetic deletion.

Supplementary Figure 5. TNF-α-induced cytokine production by dying cells is maintained when TAK1 is inhibited but not deleted.

Supplementary Figure 6. *Tak1*-deficiency does not alter the frequency or activation of tumor infiltrating lymphocytes.

Supplementary Figure 7. Gating strategy to identify and phenotype tumor infiltrating lymphocytes from CT-26 tumors.

Supplementary Figure 1A, Refers to Figure 1

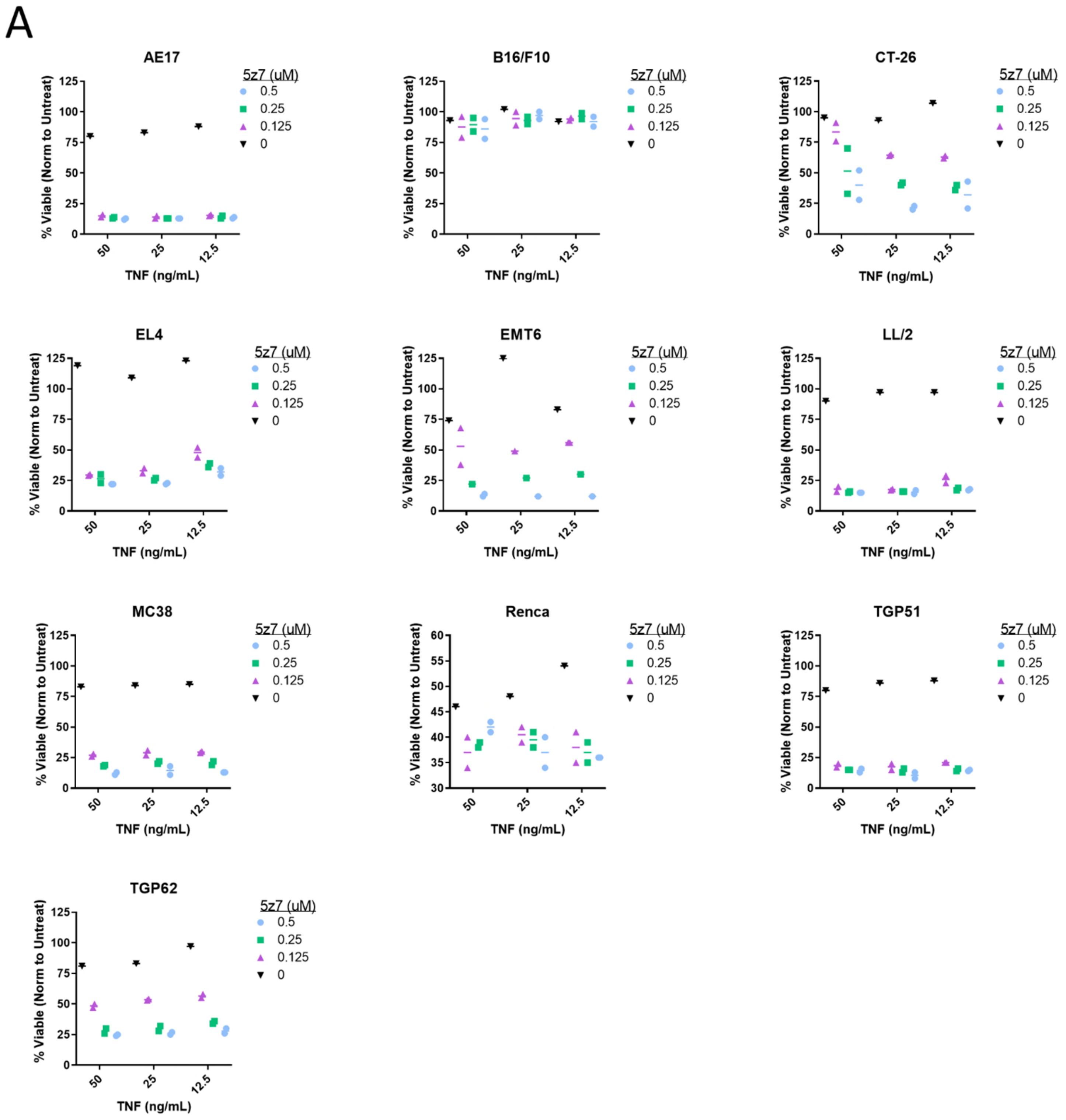

Supplementary Figure 1B, Refers to Figure 1

B

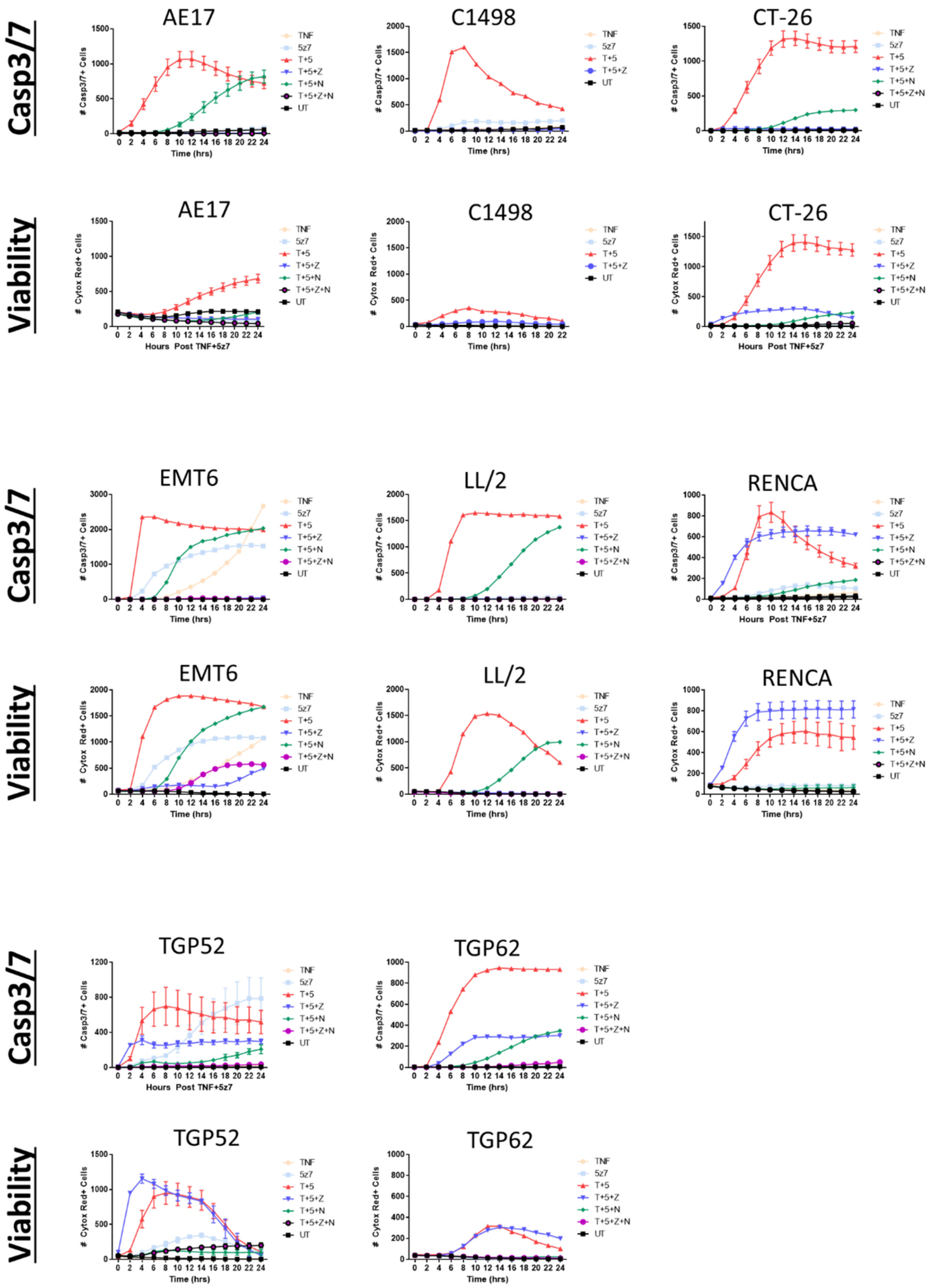

Supplementary Figure 1. The TAK1 inhibitor 5z7-oxozeaenol sensitizes mouse tumor cell lines to RIPK1 kinase-dependent, TNF-α **-mediated cytotoxicity.** (A) Mouse tumor cell lines were treated overnight with the indicated concentrations of TNF +/- 5z7-oxozeaenol (5z7) and viability was assessed via CellTiter-Glo. Data represent a single experiment, with the bar depicting the mean of duplicate measurements. (B) The indicated mouse tumor cell line was pretreated with the pan-caspase inhibitor zVAD-fmk (Z, 20 μM, 2 hrs), the RIPK1 kinase inhibitor necrostatin-1 (N, 30 μM, 1 hr), or both (Z+N) and subsequently treated with TNF-α (T, 25 ng/mL) and the TAK1 inhibitor 5z7-oxozeaenol (5, 0.125 μM). Caspase-3/-7 activity and membrane permeabilization were monitored. Data represent the mean +/- SD of a representative experiment, n=4 independent experiments.

Supplementary Figure 2, Refers to Figure 2

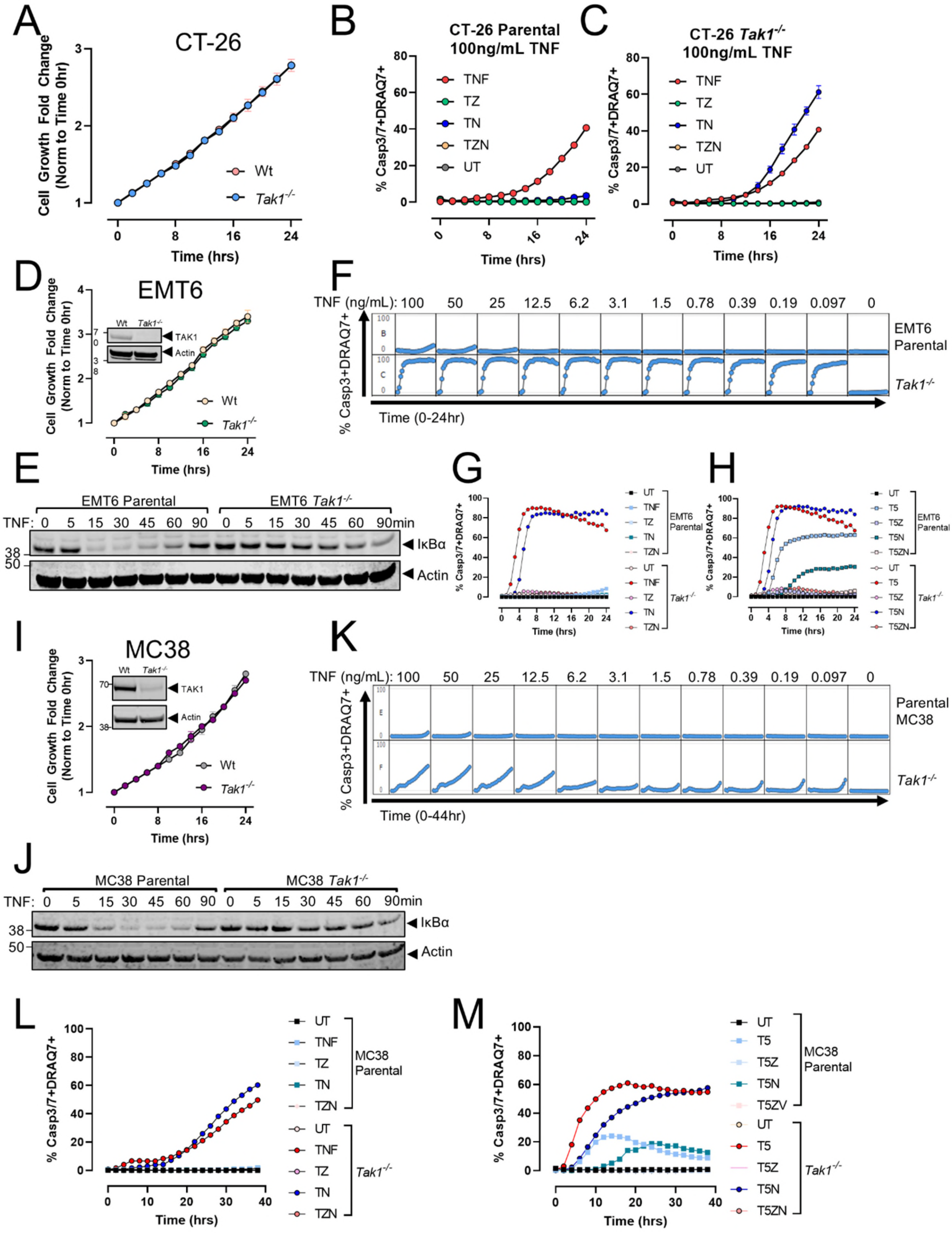

Supplementary Figure 2. Characterization of *Tak1*-deficient murine tumor cell lines. (A) In vitro growth kinetics of parental and *Tak1*$^{-/-}$ CT-26 cells assessed for 24 hrs and presented as fold change in confluency normalized to time 0 hr. (B, C) Parental (B) or *Tak1*-deficient (C) CT-26 cells were pretreated for 2hrs with zVAD-fmk (Z, 20 μM), necrostatin-1 (N, 30 μM), or both and subsequently treated with 100 ng/mL TNF-α (T). Caspase activity was assessed every 2 hrs for 48 hrs. Note how necrostatin-1 fails to rescue TNF-α -treated *Tak1*-deficient CT-26 cells. (D) In vitro growth kinetics of parental and *Tak1*$^{-/-}$ EMT6 cells assessed for 24 hrs and presented as fold change in confluency normalized to time 0 hr. Inset depicts *Tak1* knockout efficiency in EMT6 cells. (E) *Tak1*$^{-/-}$ EMT6 cells were treated with 25 ng/mL of TNF-α for the indicated time and degradation of IκBα was assessed. (F) *Tak1*$^{-/-}$ EMT6 cells were treated with the indicated concentration of TNF-α and assessed for capsase-3/-7 activity and membrane permeabilization over the duration of 24 hrs, capturing images every 2 hrs. (G, H) Parental and *Tak1*$^{-/-}$ EMT6 cells were pretreated with zVAD-fmk (Z, 20 μM), necrostatin-1 (N, 30 μM), or both and subsequently treated with (G) TNF-α (T, 25 ng/mL) or (H) TNF-α and 5z7-oxo (5, 0.125 μM). Caspase-3/-7 activity was assessed every 2 hrs for 24 hrs. (I) In vitro growth kinetics of parental and *Tak1*$^{-/-}$ MC388 cells assessed for 24 hrs and presented as fold change in confluency normalized to time 0 hr. Inset depicts *Tak1* knockout efficiency in MC38 cells (J) *Tak1*$^{-/-}$ MC38 cells were treated with 25ng/mL of TNF-α for the indicated time and degradation of IκBα was assessed. (K) *Tak1*$^{-/-}$ MC38 cells were treated with the indicated concentration of TNF-α and assessed for capsase-3/-7 activity and membrane permeabilization over the duration of 44hrs, capturing images every 2 hrs. (L, M) Parental *Tak1*$^{-/-}$ MC38 cells were pretreated with zVAD-fmk (Z, 20 μM), necrostatin-1 (N, 30 μM), or both and subsequently treated with (L) TNF-α (T, 25 ng/mL) or (M) TNF-α and 5z7-oxo (5, 0.125 μM). Caspase-3/-7 activity was assessed every 2 hrs for 40 hrs. For IncuCyte experiments, data represent the mean -/+ SD of an individual experiment, n = 3 independent experiments. Western blots are representative of two independent experiments. Data in F and K are the mean from 4 imaged areas of a representative experiment from n = 3 independent experiments.

Supplementary Figure 3, Refers to Figure 2

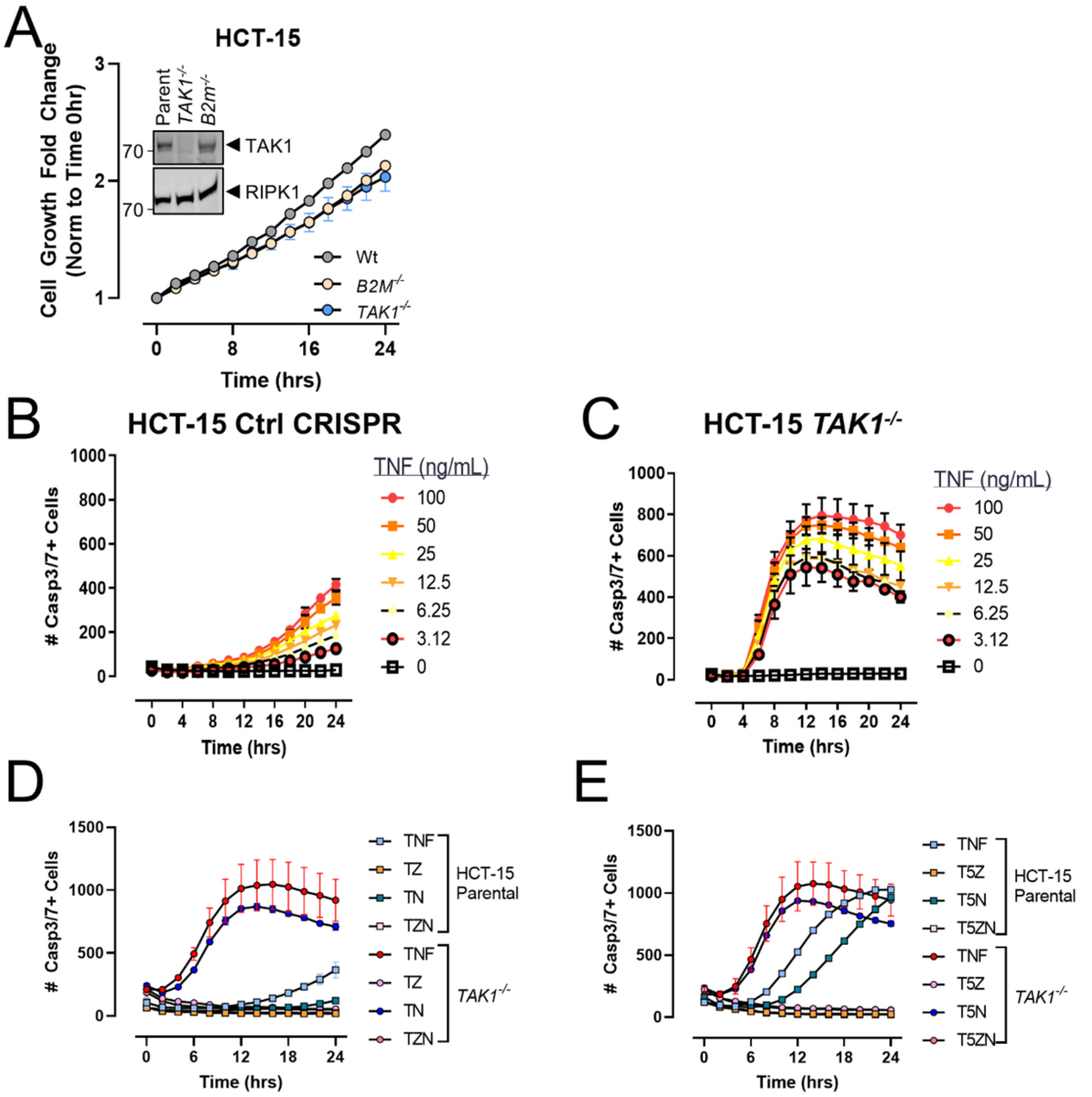

Supplementary Figure 3. Characterization of *TAK1*-deficient HCT-15 cells. (A) *TAK1* deletion in HCT-15 cells does not alter growth kinetics. Inset depicts *Tak1* knockout efficiency in HCT-15 cells. Cell proliferation was assessed for 24 hrs and presented as confluency fold change normalized to time 0hr. Note that although the *TAK1*-defienct clone grows slightly slower than the parental line, it exhibits comparable growth to the control knockout clone (*B2M*$^{-/-}$). (B, C) HCT-15 control CRISPR (B) or *TAK1*$^{-/-}$ (C) cells were treated with the indicated dose of TNF-α. The number of caspase-3/-7+ cells was assessed every 2 hrs for 24 hrs. (D, E) Parental or *TAK1*$^{-/-}$ HCT-15 cells were pretreated with zVAD-fmk (Z, 20 μM), necrostatin-1 (N, 30 μM), or both and subsequently treated with (D) TNF-α (T, 25 ng/mL) or (E) TNF-α and 5z7-oxo (5, 0.125 μM). Caspase-3/-7 activity was assessed every 2 hrs for 24 hrs. Data represent the mean -/+ SD of an individual experiment, n = 2-3 independent experiments.

Supplementary Figure 4, Refers to Figure 2

A

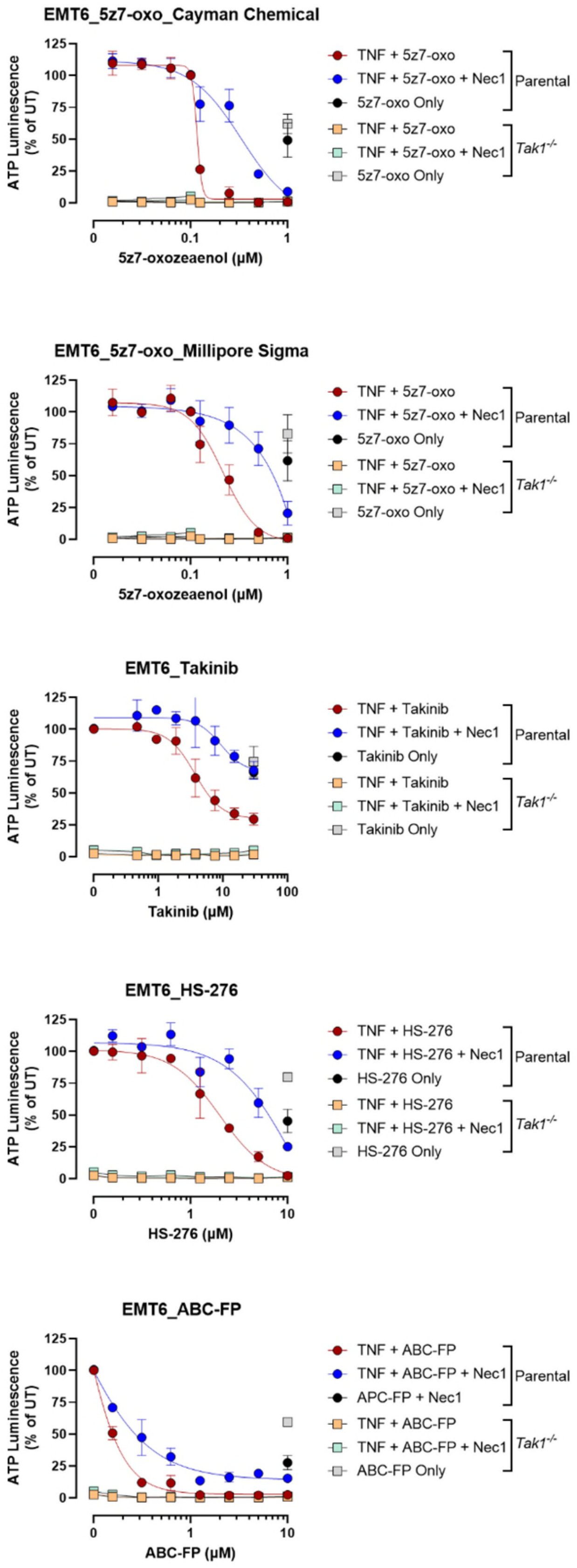

B

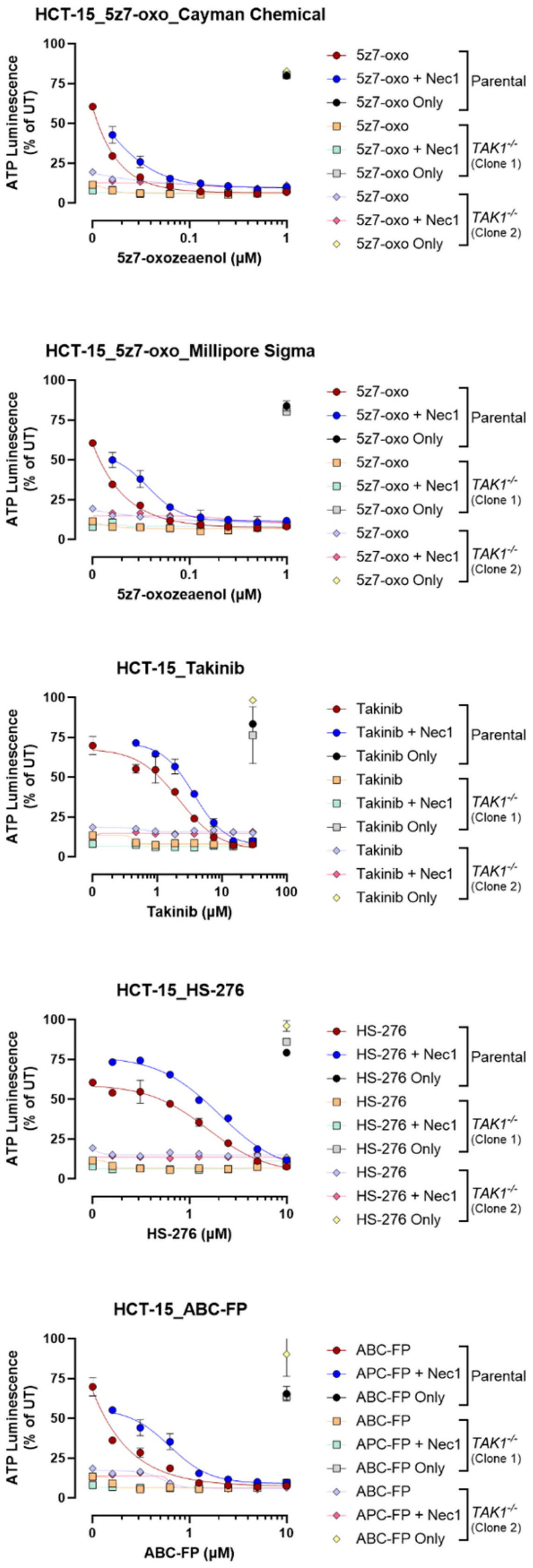

Supplementary Figure 4. Differential requirement for RIPK1 kinase activity during TNF-mediated cell death in tumor cells upon TAK inhibition vs genetic deletion. (A, B) Parental or TAK1-deficient tumor cells (A, EMT6; B, HCT-15) were treated overnight with TNF-α (12.5ng/mL) and the indicated TAK1 inhibitor in the presence or absence of necrostatin-1 (30μM). Cell death was assessed by Cell Titer glo. Data represent the mean -/+ SD of an individual experiment, n = 3 independent experiments.

Supplementary Figure 5, Refers to Figure 3

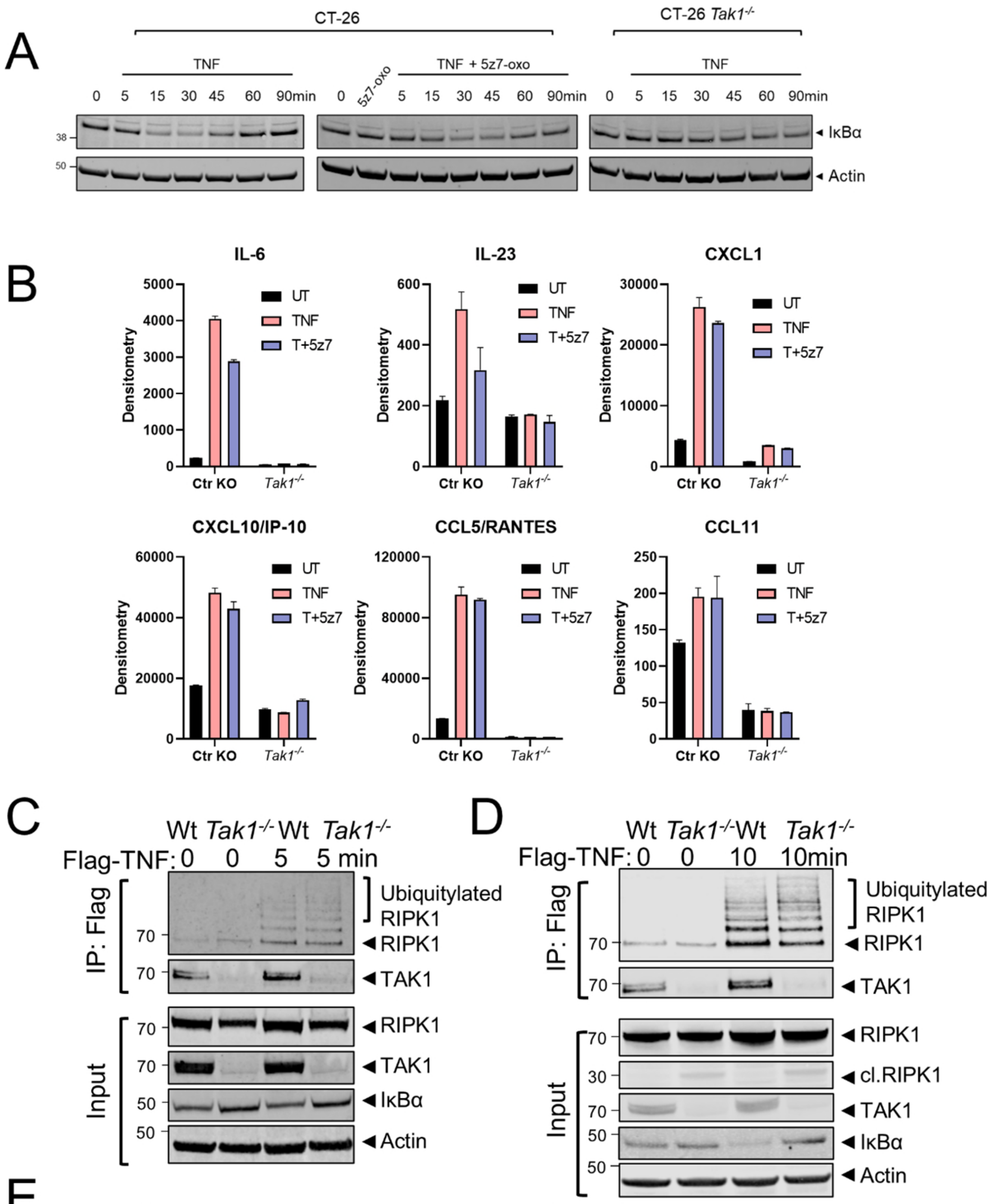

E

	RNA-seq (FPKM)		
	CT-26(GSE247046)	MC38(GSE247046)	EMT6(GSE217132)
Tnfrsf1a	61.12	40.75	54.73
Casp8	13.35	22.29	19.71
Tradd	11.38	8.91	12.91
Fadd	7.70	6.61	2.45
Ripk1	16.37	12.25	8.81
Ripk3	0.02	3.87	0.00
Cflar	100.11	13.08	16.17

Supplementary Figure 5. NF-kB pathway activation and cytokine production coincide with TNF-α-mediated cytotoxicity driven by TAK1 pharmacologic, but not genetic, inhibition. (A) CT-26 parental and *Tak1*$^{-/-}$ tumor cells were treated with TNF-α (12.5ng/mL) and 5z7-oxo (0.125μM) and IκBα degradation was assessed at the indicated times. Data depicts a representative result from 3 independent experiments. (B) CT-26 parental or *Tak1*$^{-/-}$ cells were treated with TNF-α +/- 5z7-oxo for 24 hrs and cytokines were measured in the supernatant. Data represent the mean +/- SD of a representative experiment, n=2 independent experiments. (C, D) Parental or *Tak1*$^{-/-}$ CT-26 cells were stimulated with FLAG-TNF-α for 5 (C) or 10 (D) minutes and complex I was immunoprecipitated using anti-FLAG agarose resin. Immunoprecipitants were probed for the indicated proteins. Note that 5 minutes is prior to NF-κB activation and 10 minutes is after pathway activation. (E) Gene expression analysis of TNFR1 complex I and II components in the indicated mouse tumor cell lines. Data were accessed from the Gene Expression Omnibus using the listed accession numbers.

Supplementary Figure 6, Refers to Figure 7

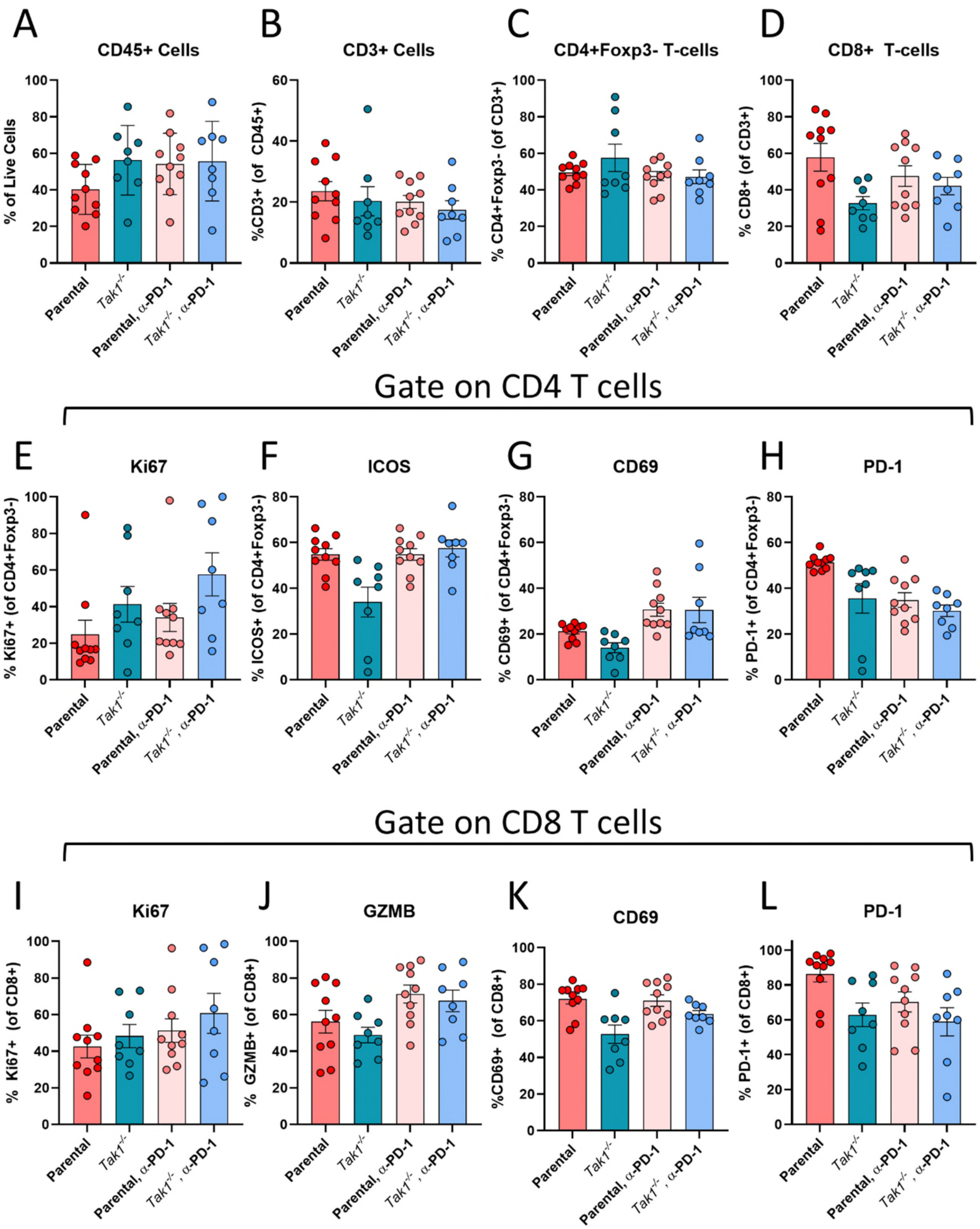

Supplementary Figure 6. *Tak1*-deficiency does not alter the frequency or activation of tumor infiltrating lymphocytes. Day 17 parental or *Tak1*$^{-/-}$ CT-26 tumors from vehicle and α-PD-1 treated Balb/c mice were harvested and assessed for tumor infiltrating lymphocytes. The frequency of (A) CD45+, (B) CD3+, (C) CD4+, or (D) CD8+ cells was assessed. CD4+ conventional T cells were defined as CD45+CD3+Foxp3- and CD8+ T cells were identified as CD45+CD3+CD8+. Gating on CD4 conventional T cells, the percentage of cells expressing (E) Ki67, (F) ICOS, (G) CD69, or (H) PD-1 was assessed. Gating on CD8 T cells, the percentage of cells expressing (I) Ki67, (J) Granzyme B (GZMB), (K) CD69, or (L) PD-1 was assessed. Data represent the mean +/- SEM of a single experiment. Each point represents an individual mouse.

Supplementary Figure 7, Refers to Figure 7

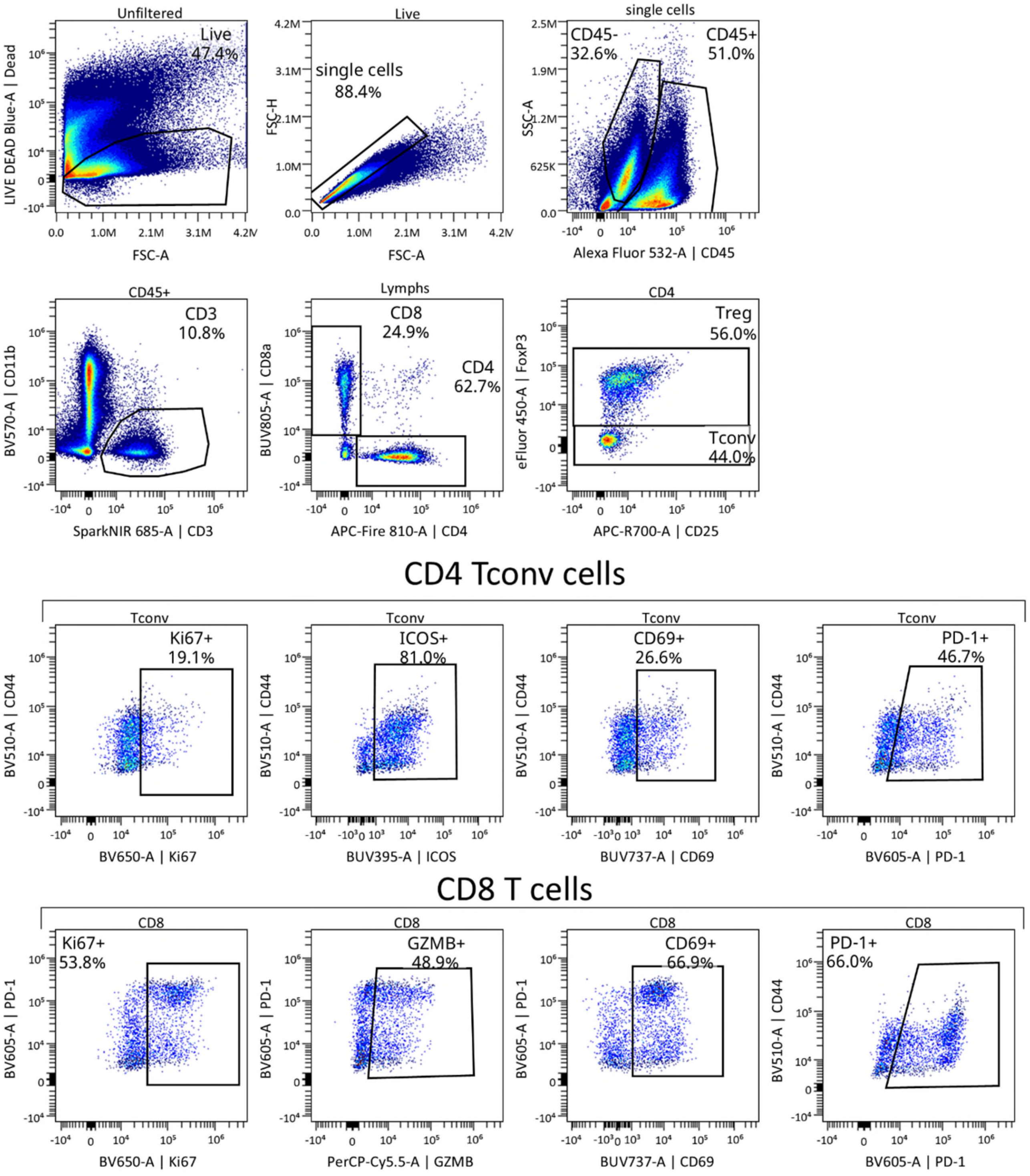

Supplementary Figure 7. Gating strategy to identify and phenotype tumor infiltrating lymphocytes from CT-26 tumors.
