# Supplementary material for: Targeting tumor intrinsic TAK1 engages TNF-α-driven cell death through distinct mechanisms and enhances cancer immunotherapy
Source: Cell Death Dis. 2025 Oct 16;16(1):725. doi: 10.1038/s41419-025-08013-0 (PMC12532812; doi:10.1038/s41419-025-08013-0)

Source Data Figure 1

A

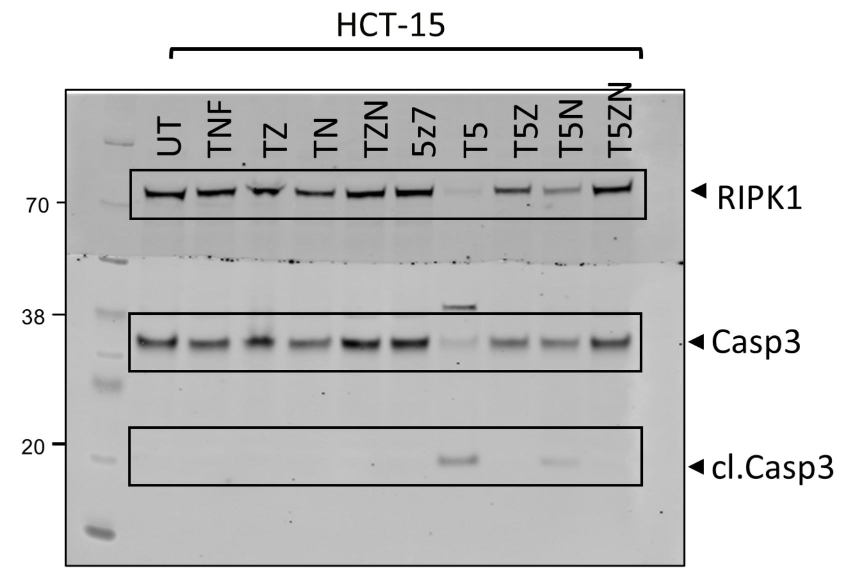

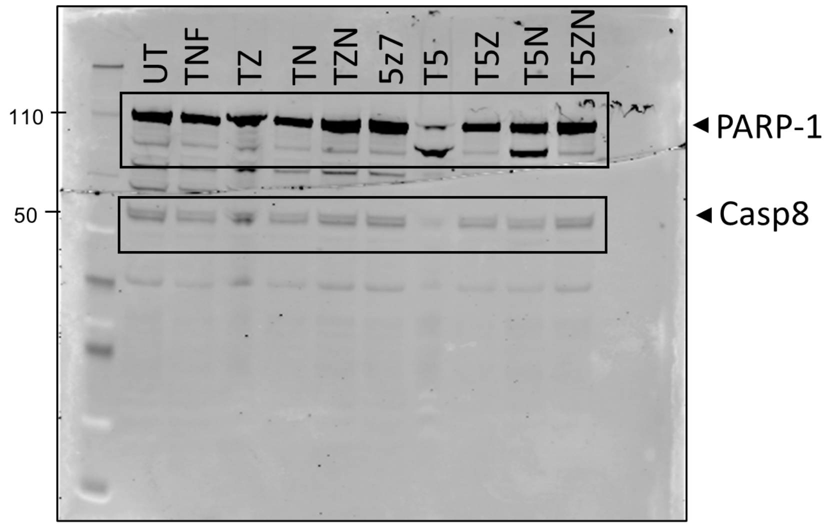

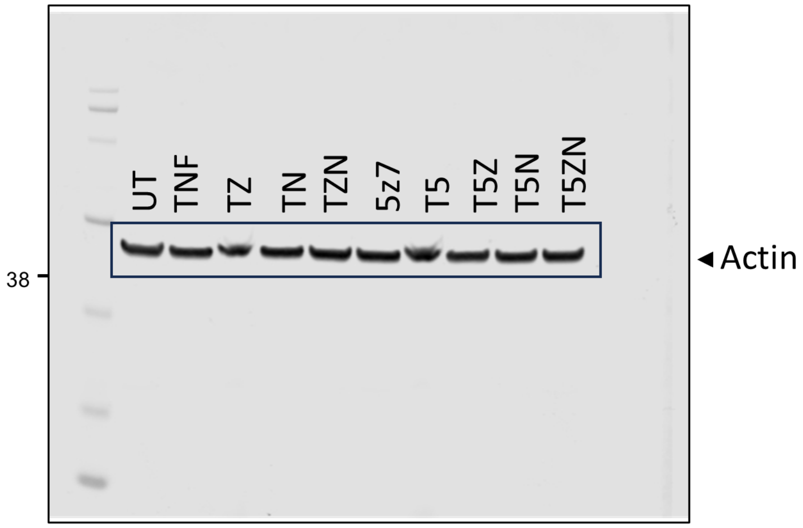

Source Data Figure 1

C

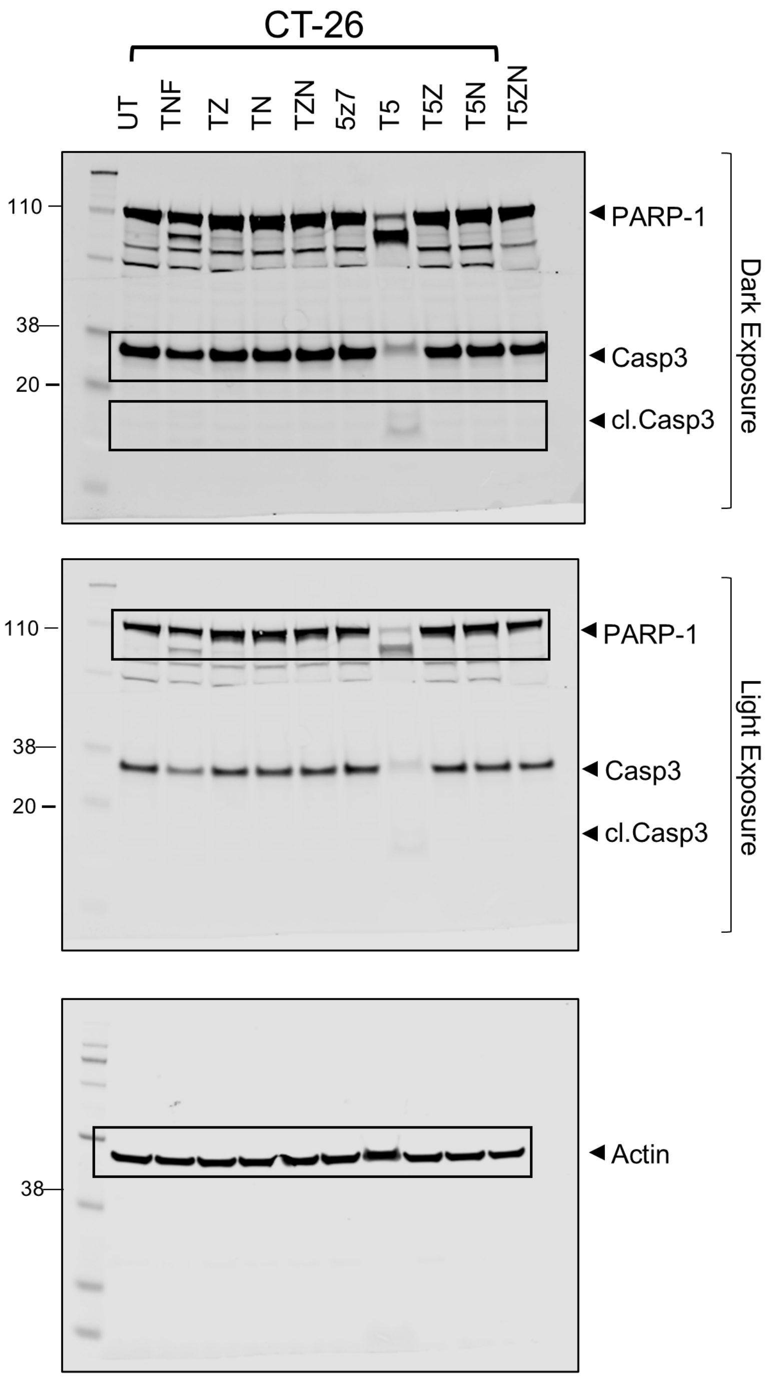

Source Data Figure 1

D

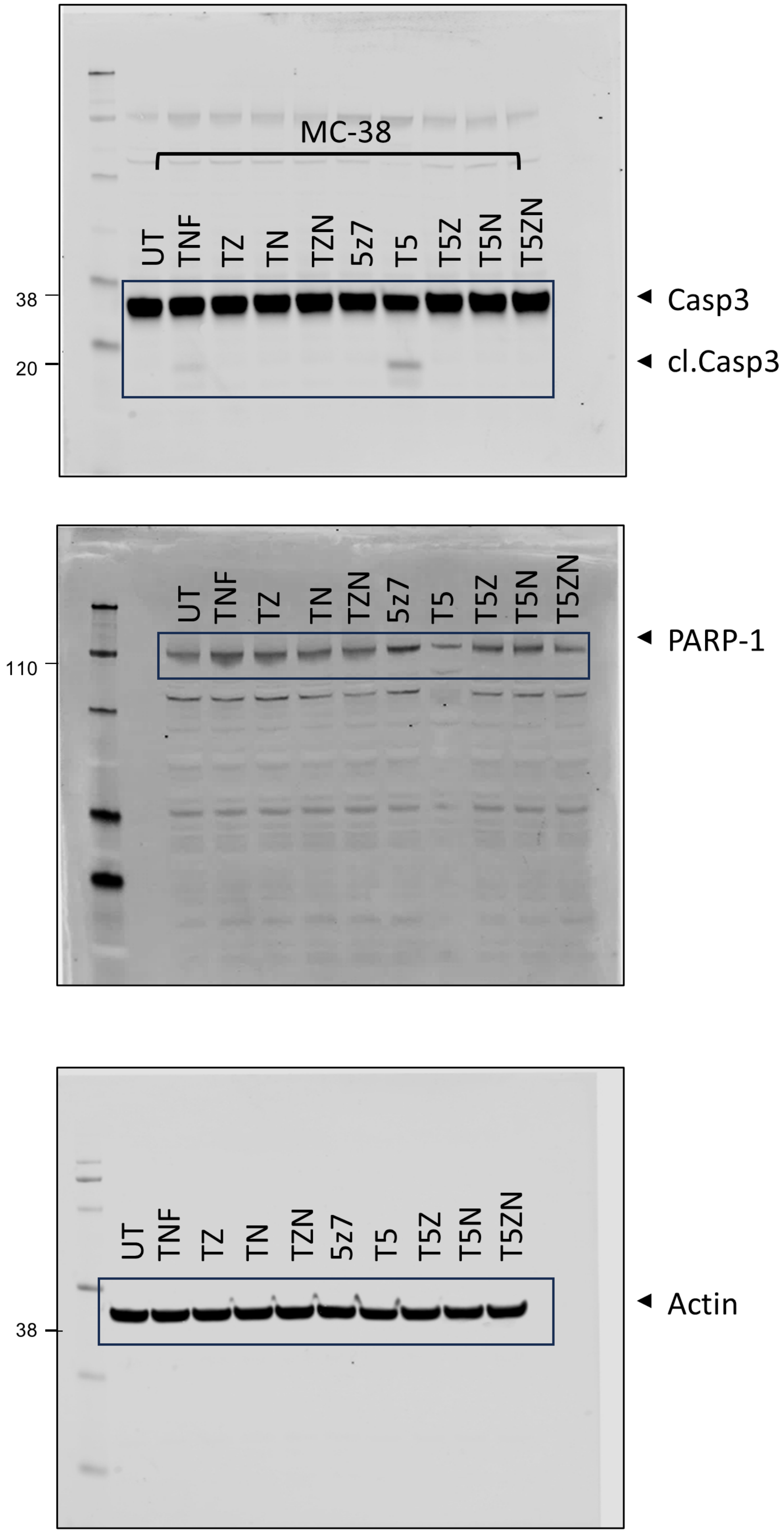

Source Data Figure 1

H, I, J

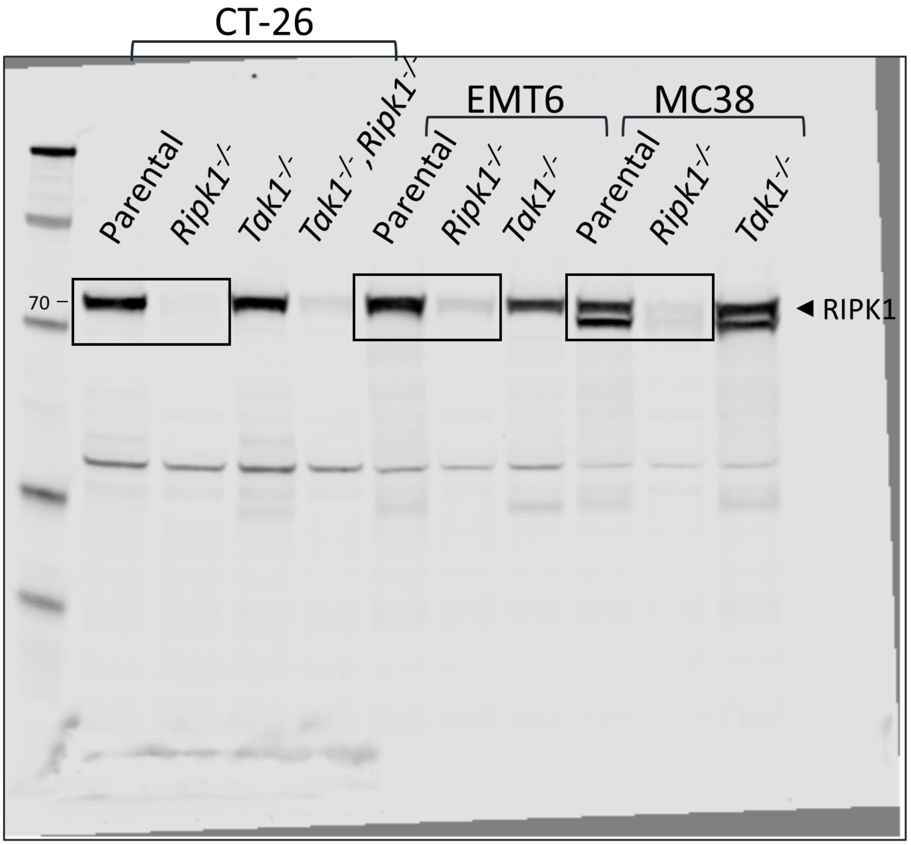

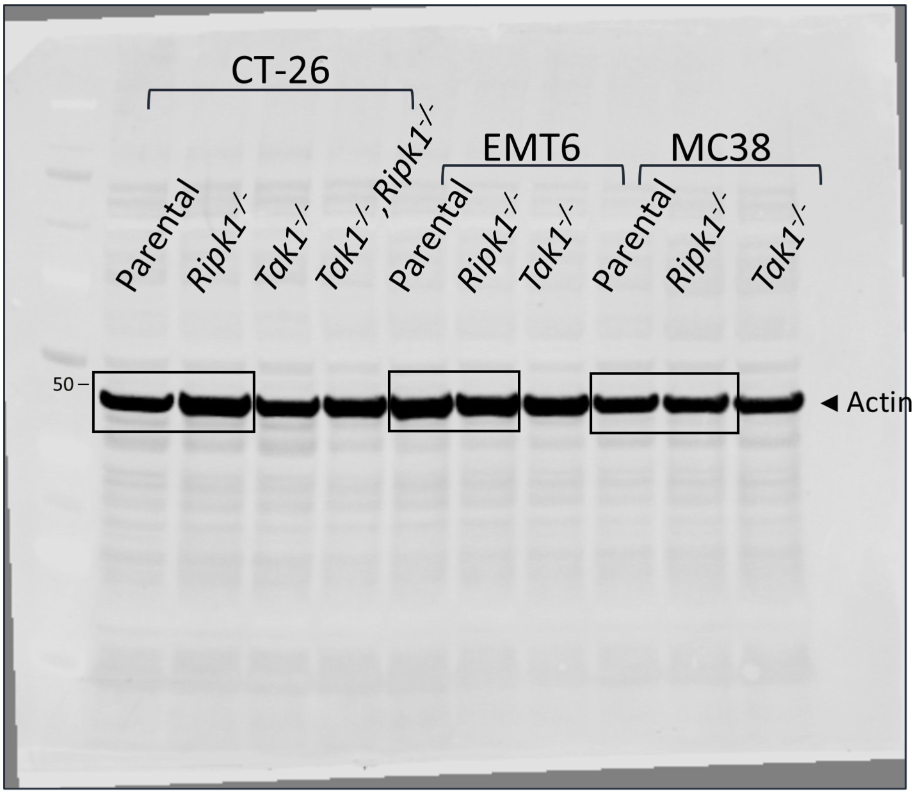

Source Data Figure 2

A

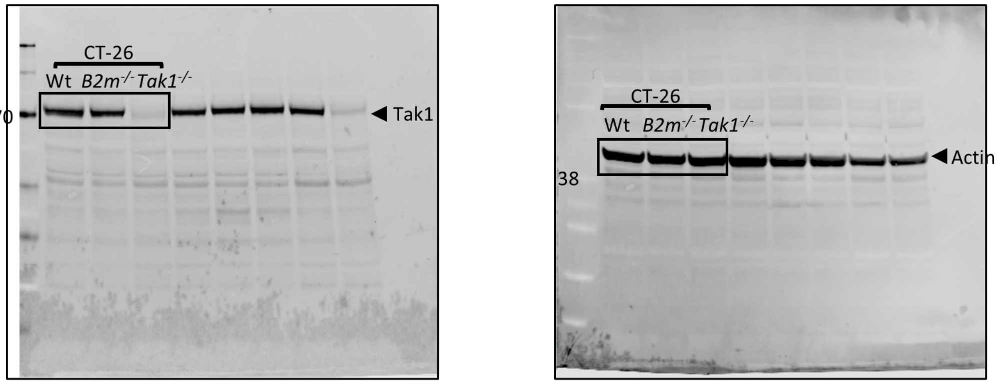

B

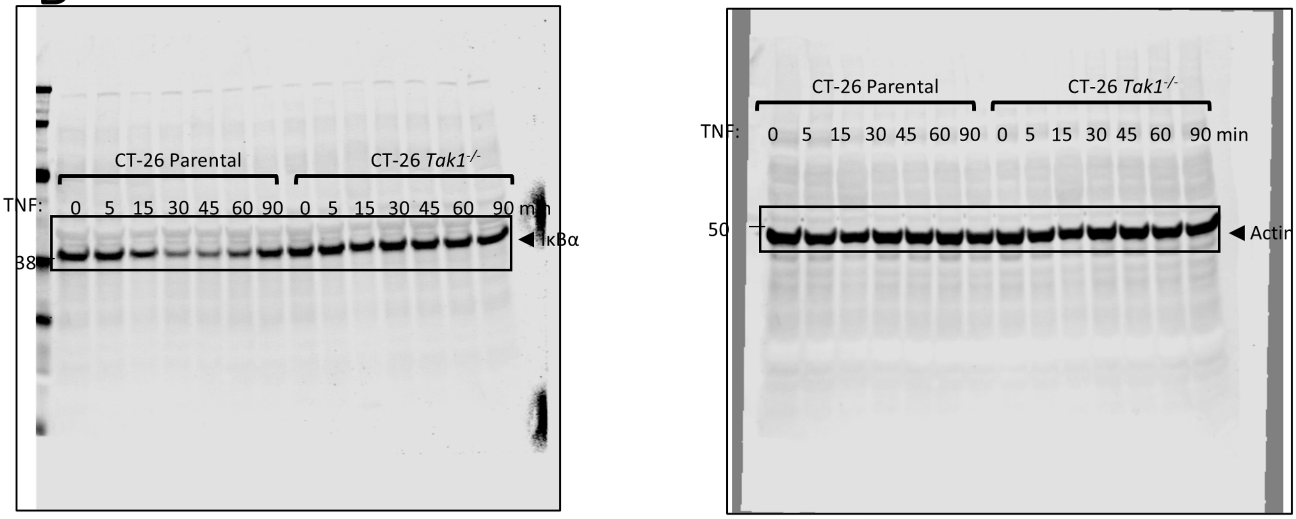

Source Data Figure 2

D

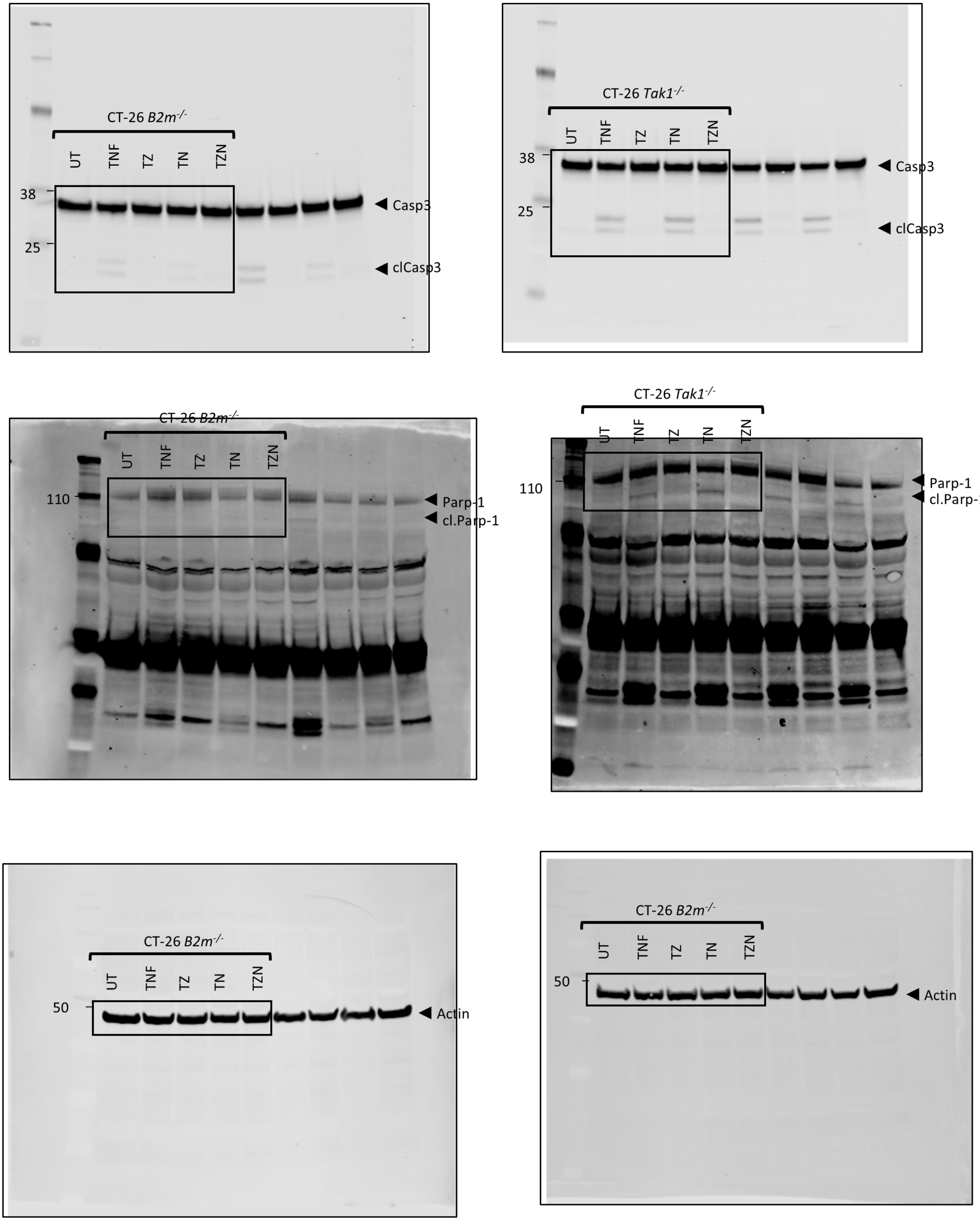

Source Data Figure 2

E

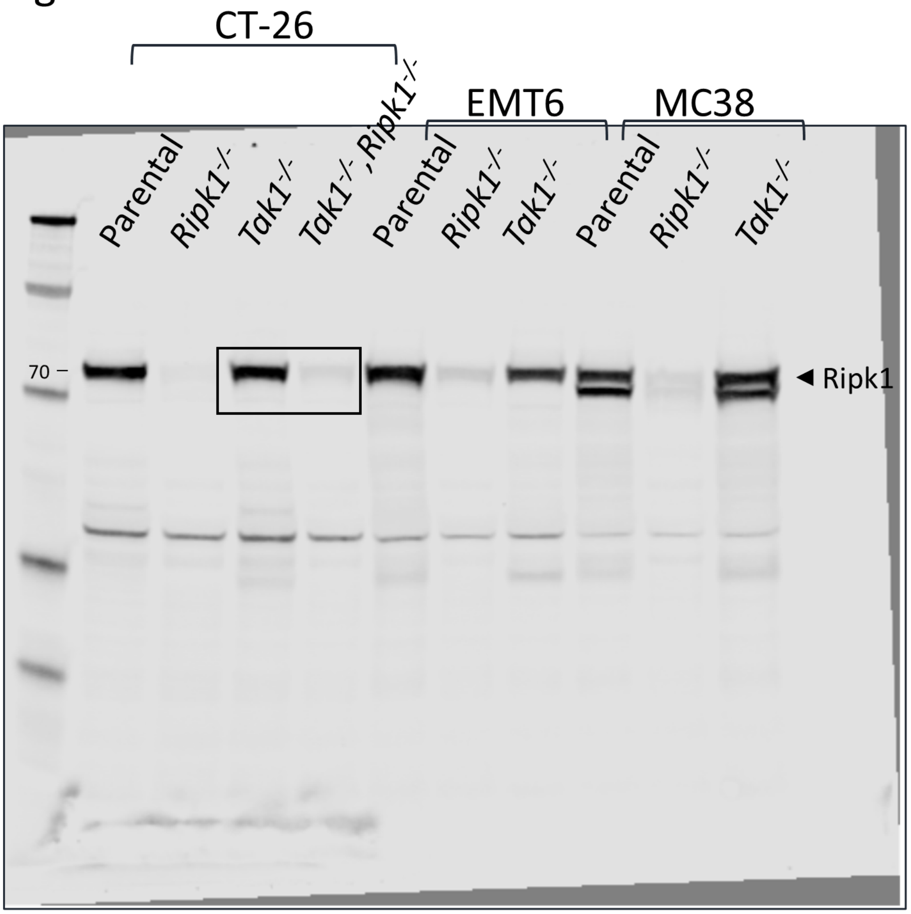

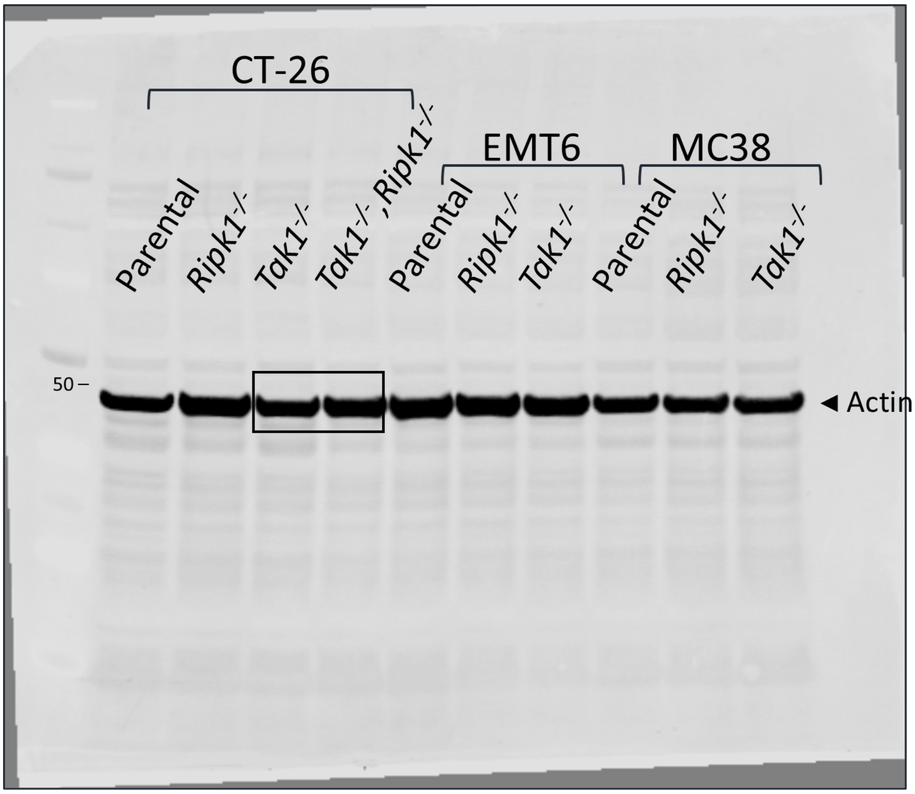

Source Data Figure 3

A

Gel 1: 1st Probe, Ripk1, 2nd Probe Caspase-8

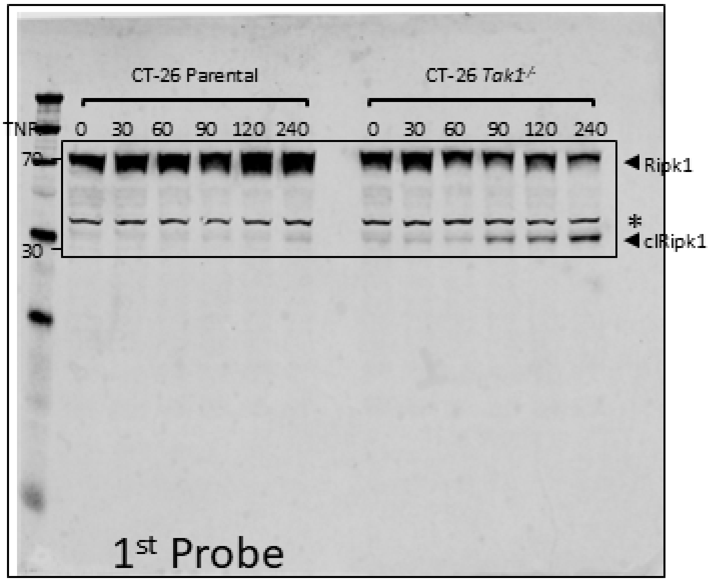

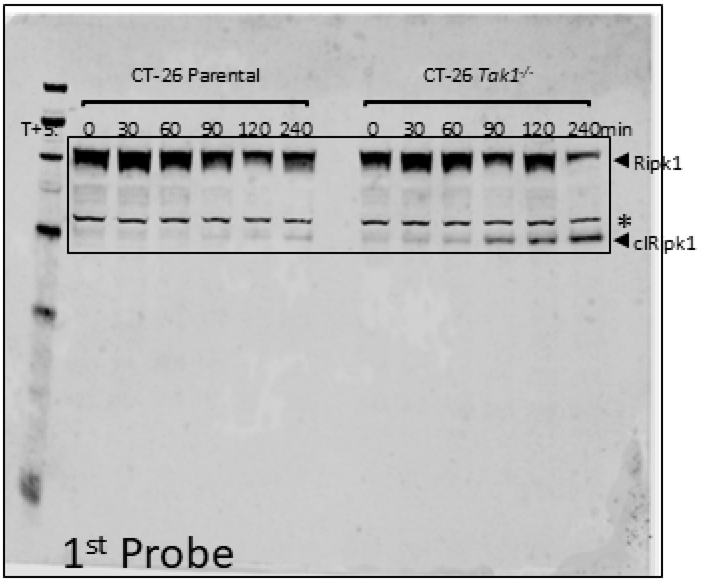

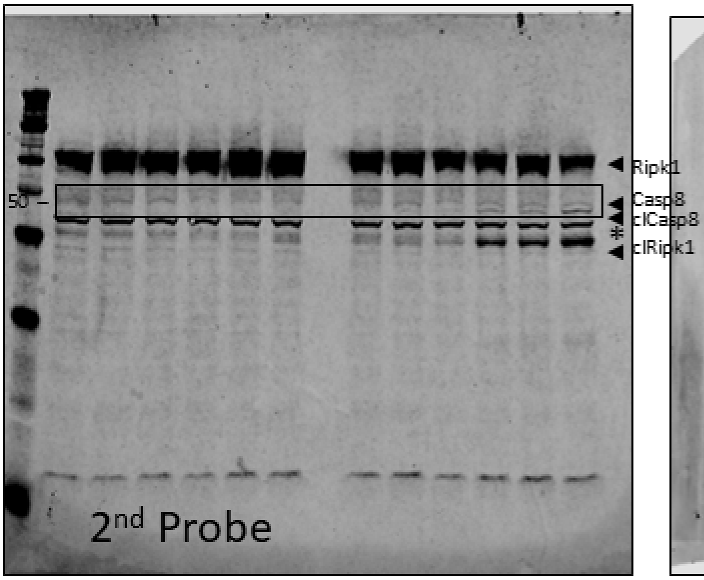

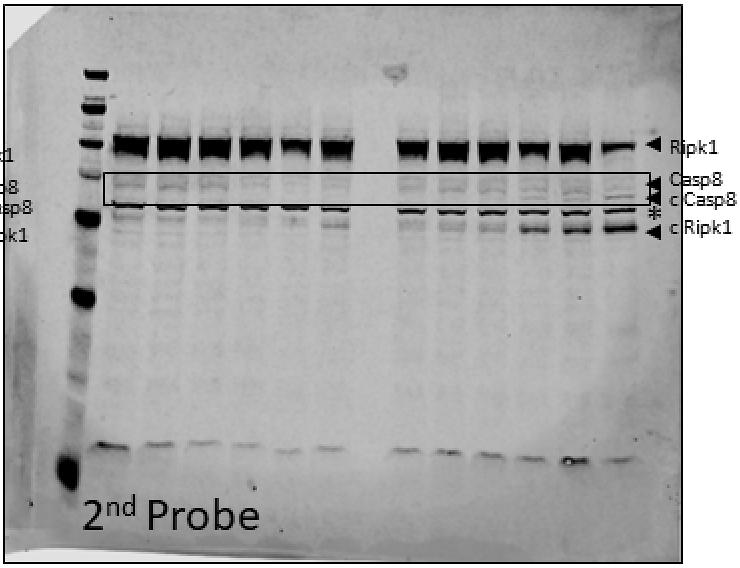

Source Data Figure 3

A

Gel 2: 1st Probe, cFlip

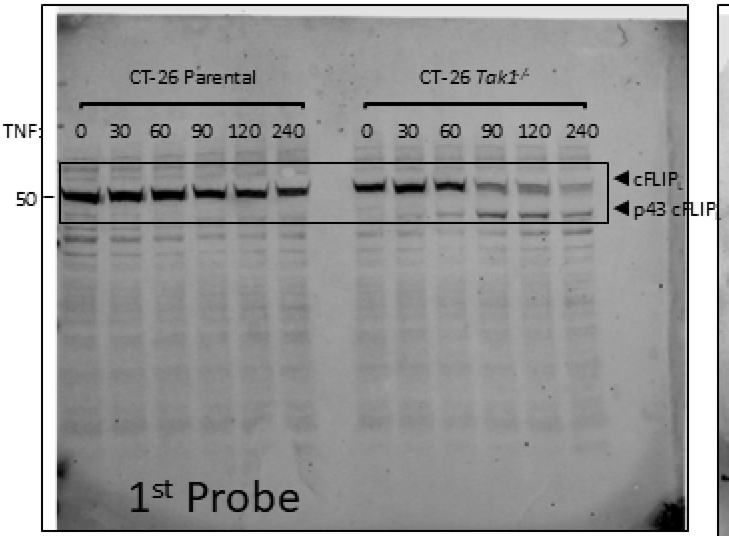

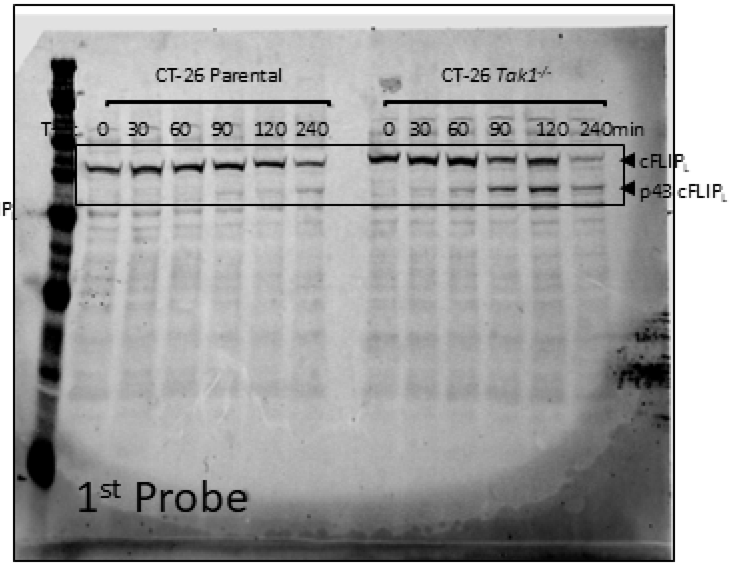

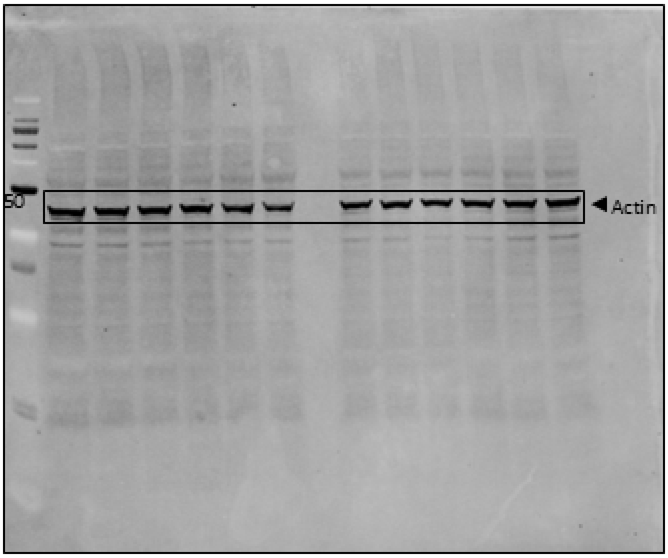

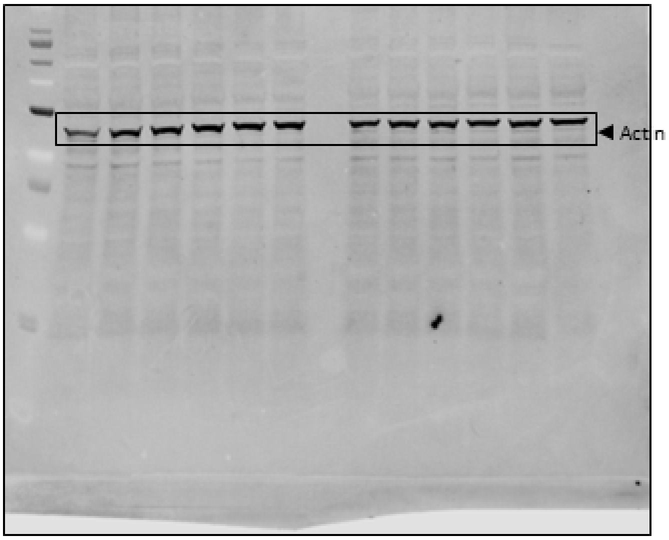

Source Data Figure 3

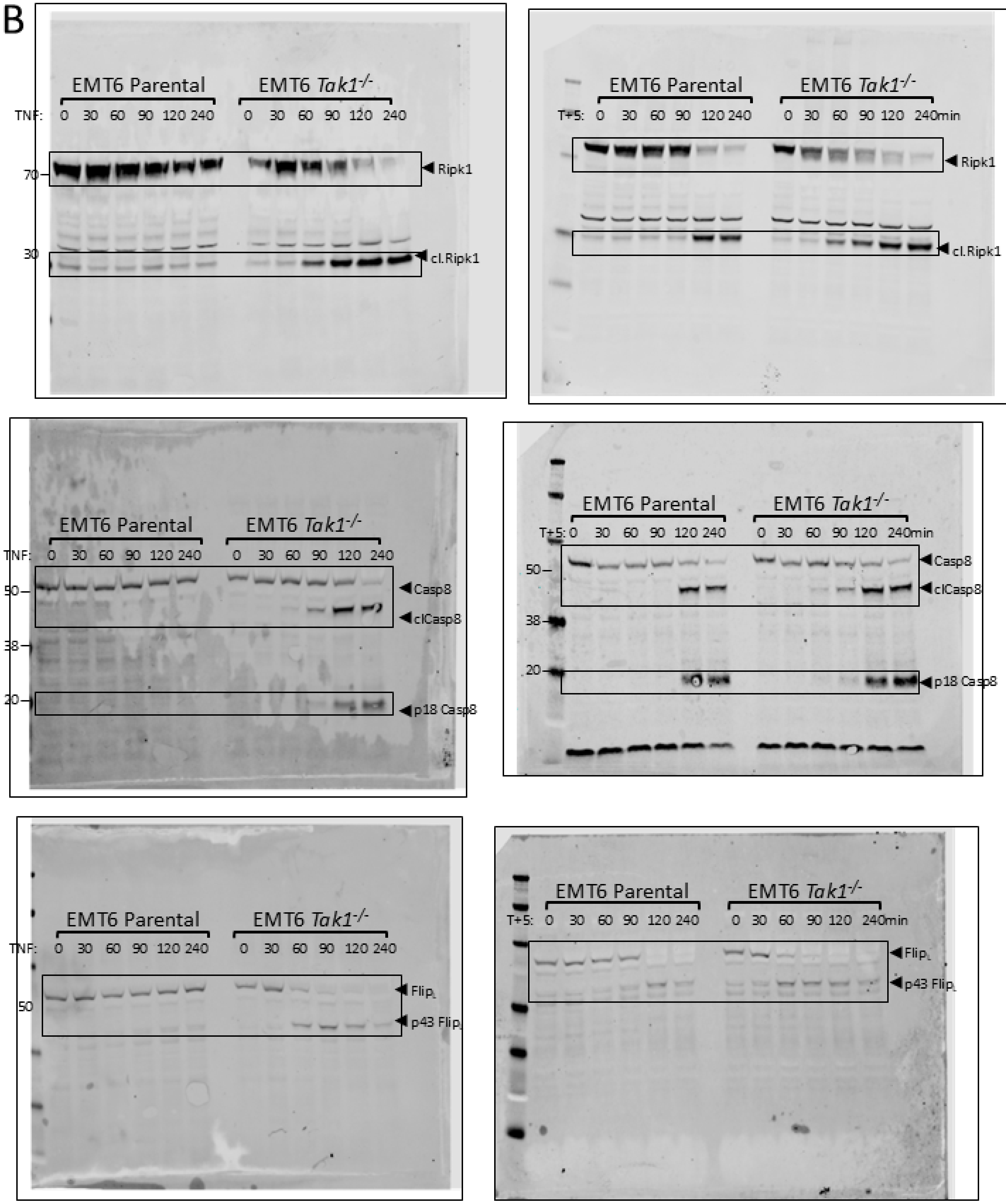

Source Data Figure 3

B

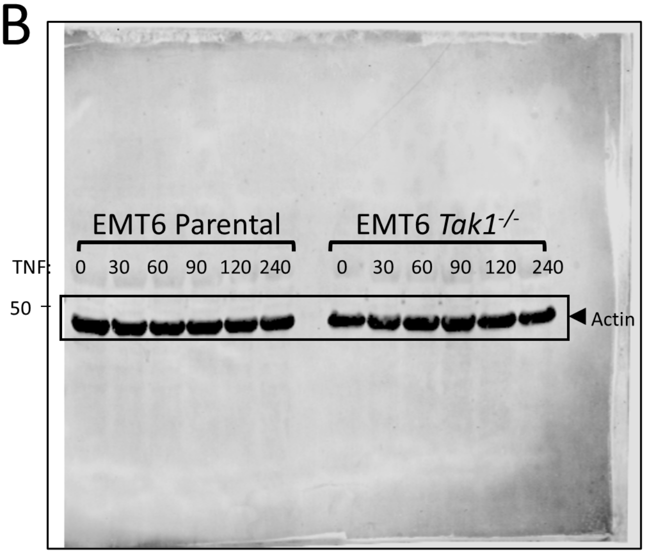

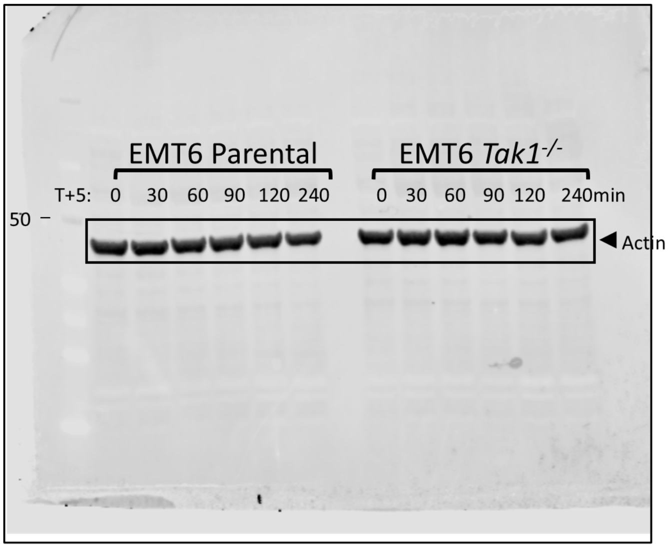

Source Data Figure 3

C Gel 1: 1st Probe, Ripk1, 2nd Probe Caspase-8

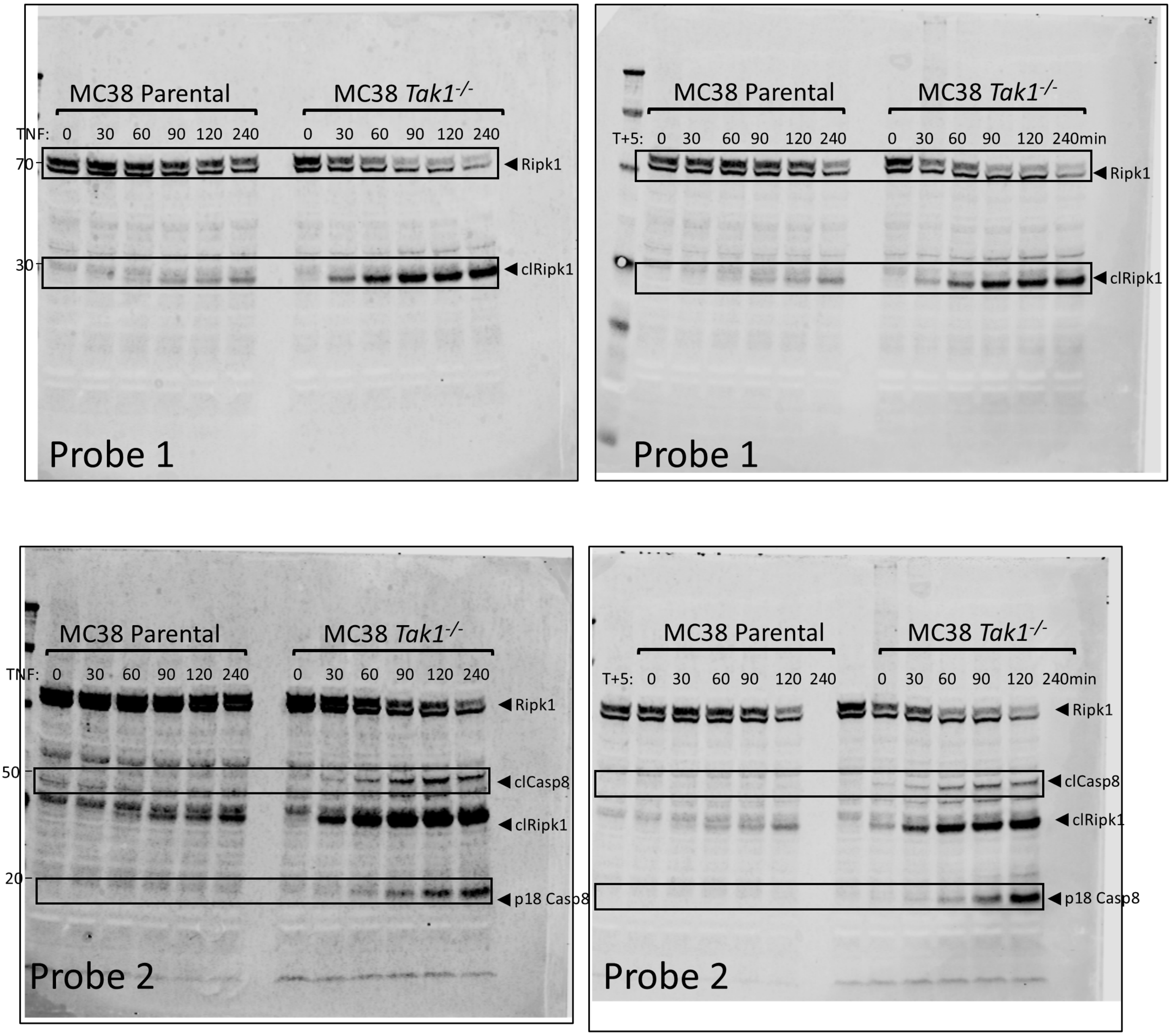

Source Data Figure 3

C Top of blot cut off for unrelated experiment

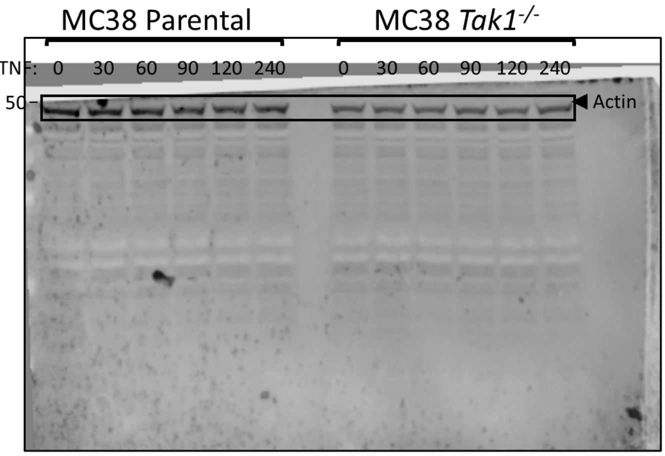

Top of blot cut off for unrelated experiment

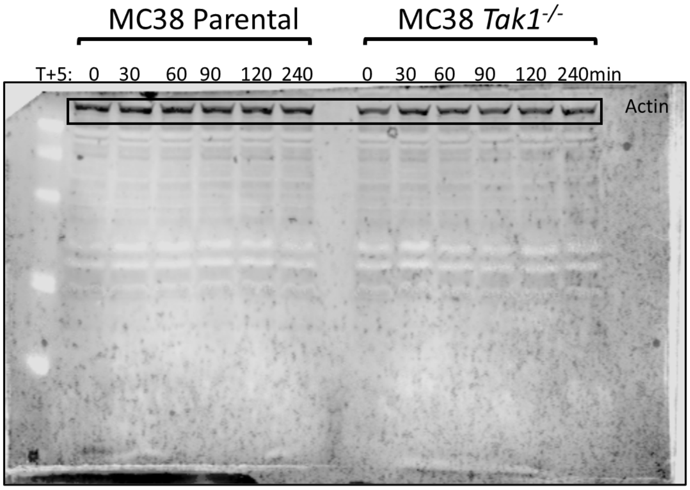

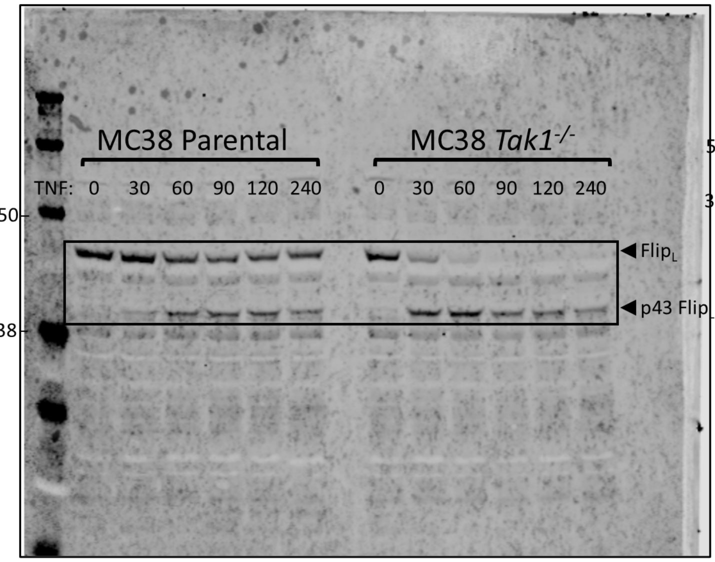

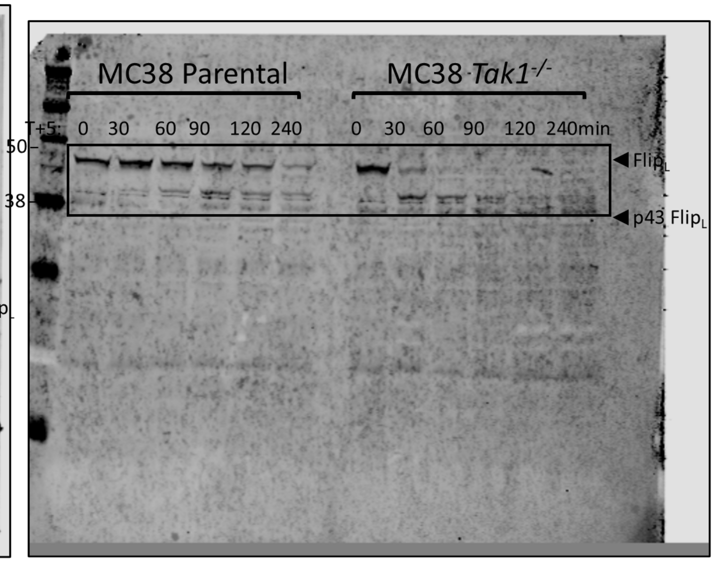

Source Data Figure 3

D

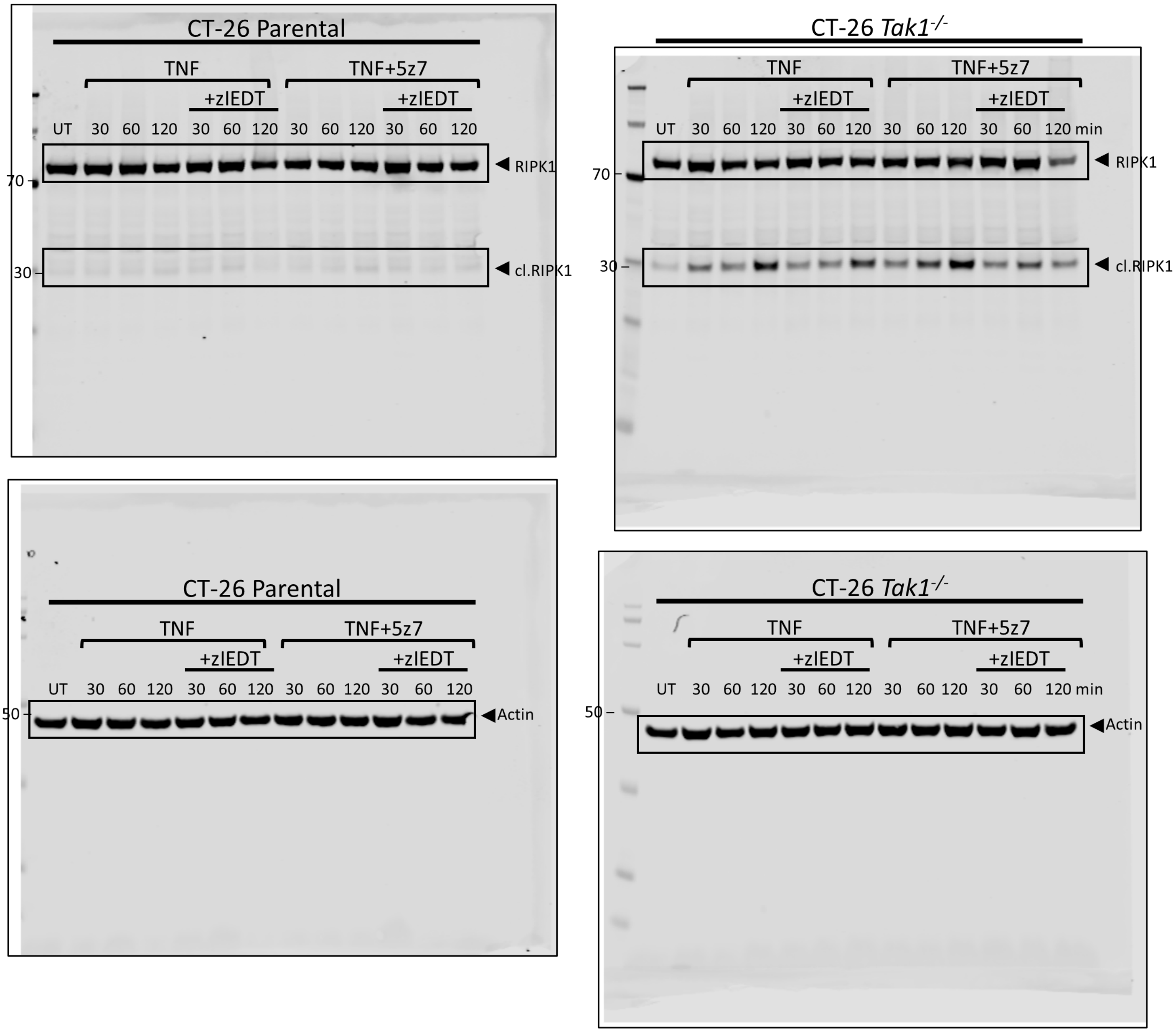

Source Data Figure 4

A

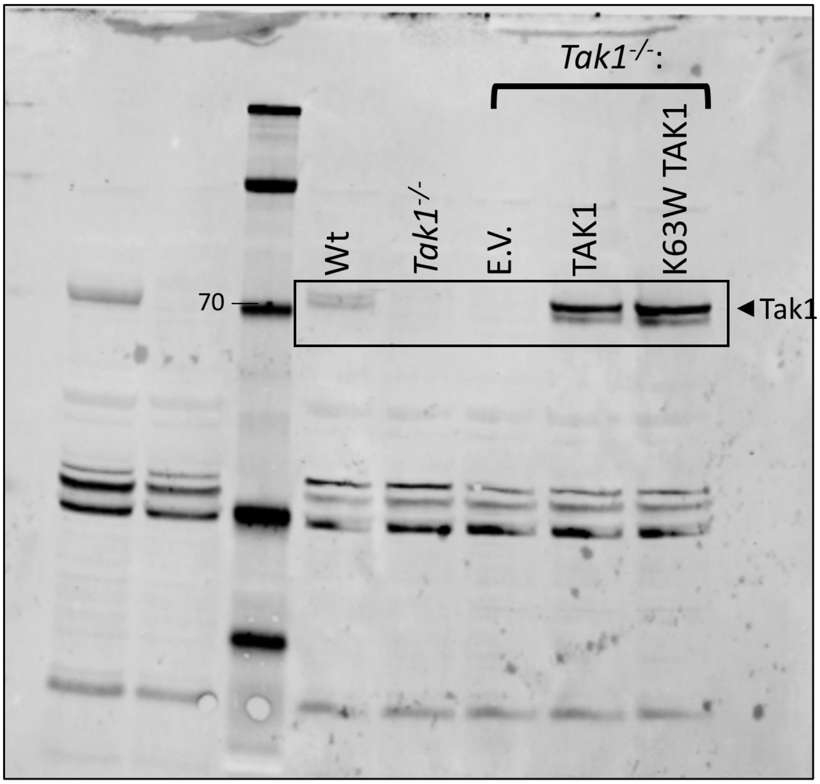

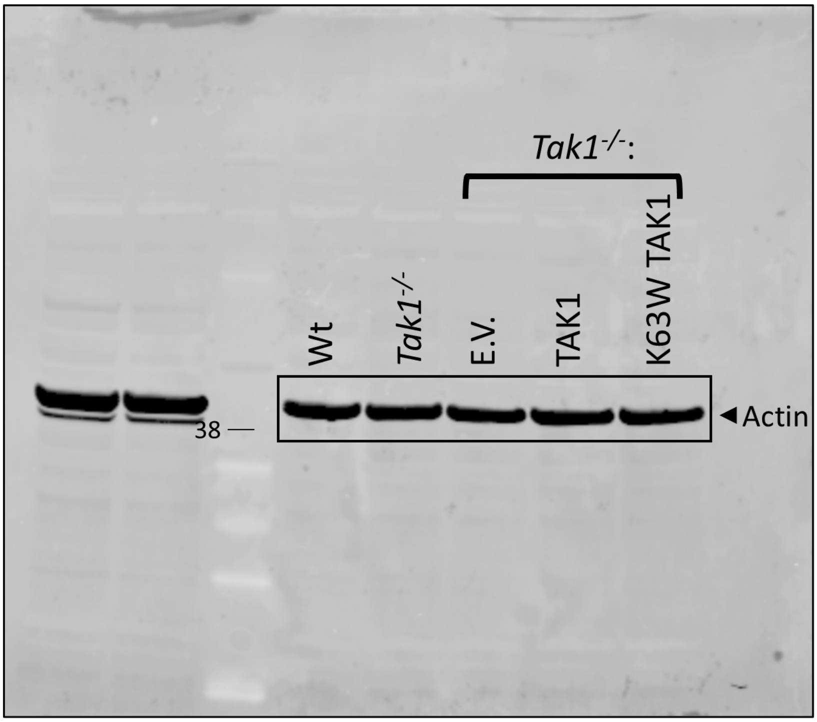

Source Data Supplementary Figure 2

D

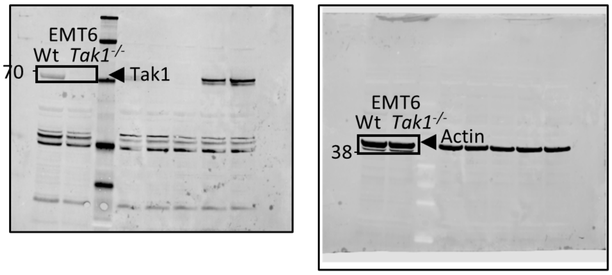

E

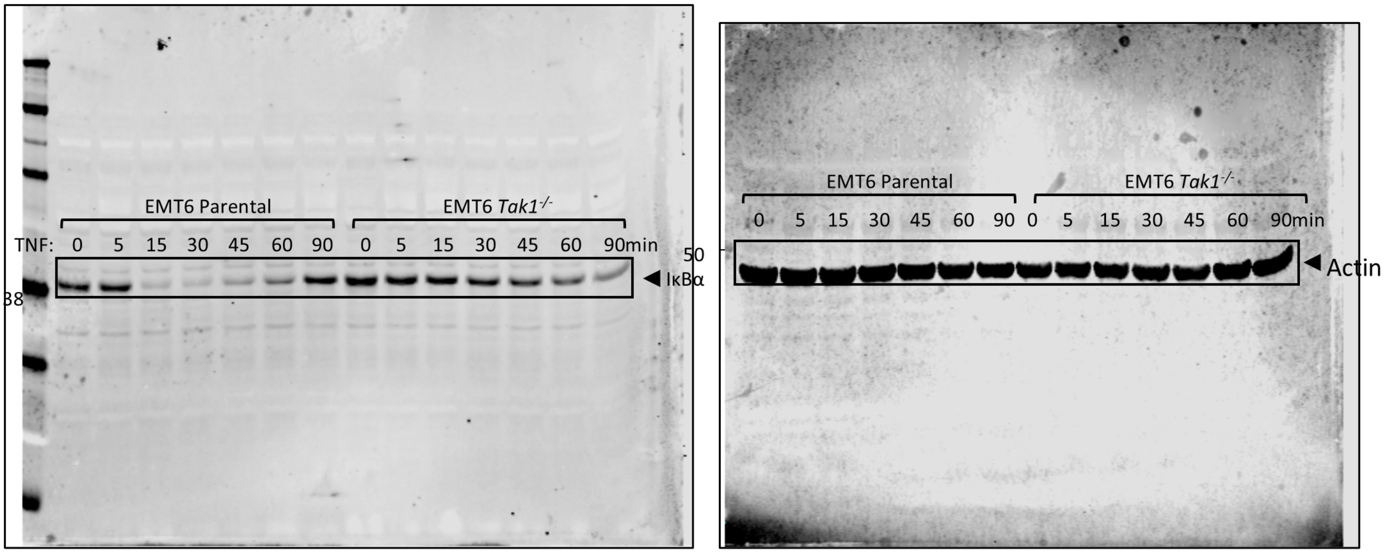

I

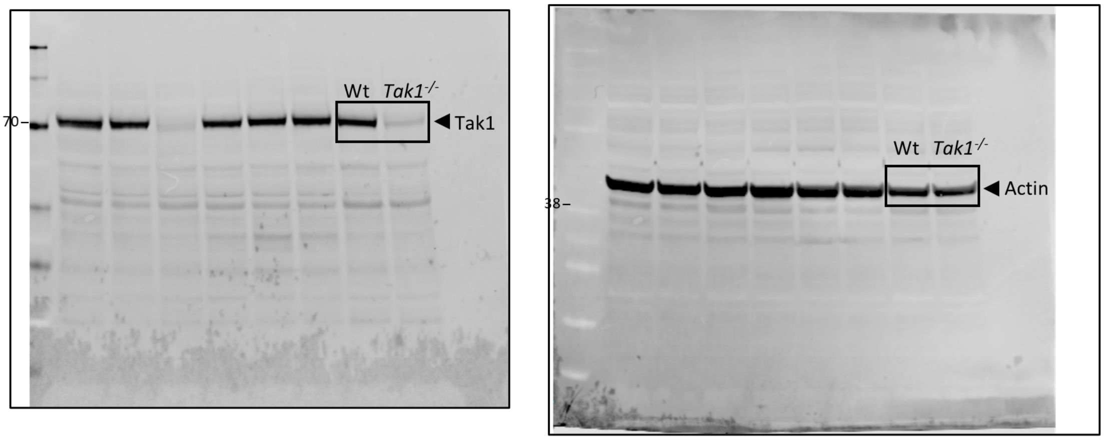

J

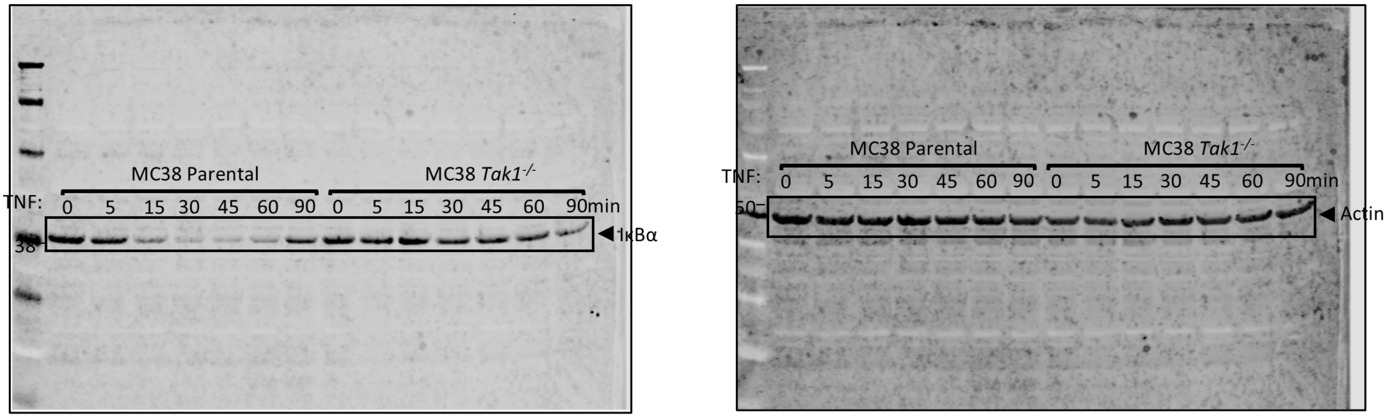

Source Data Supplementary Figure 3

A

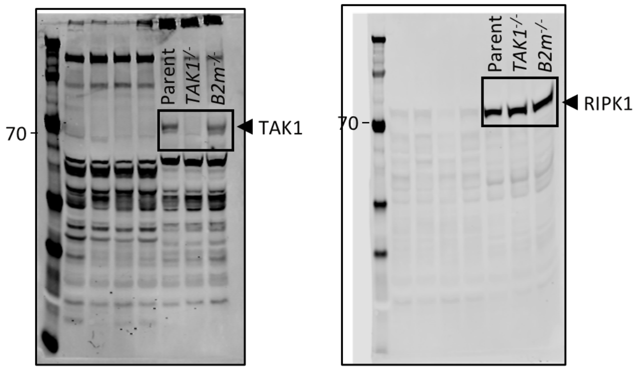

Source Data Supplementary Figure 5

A

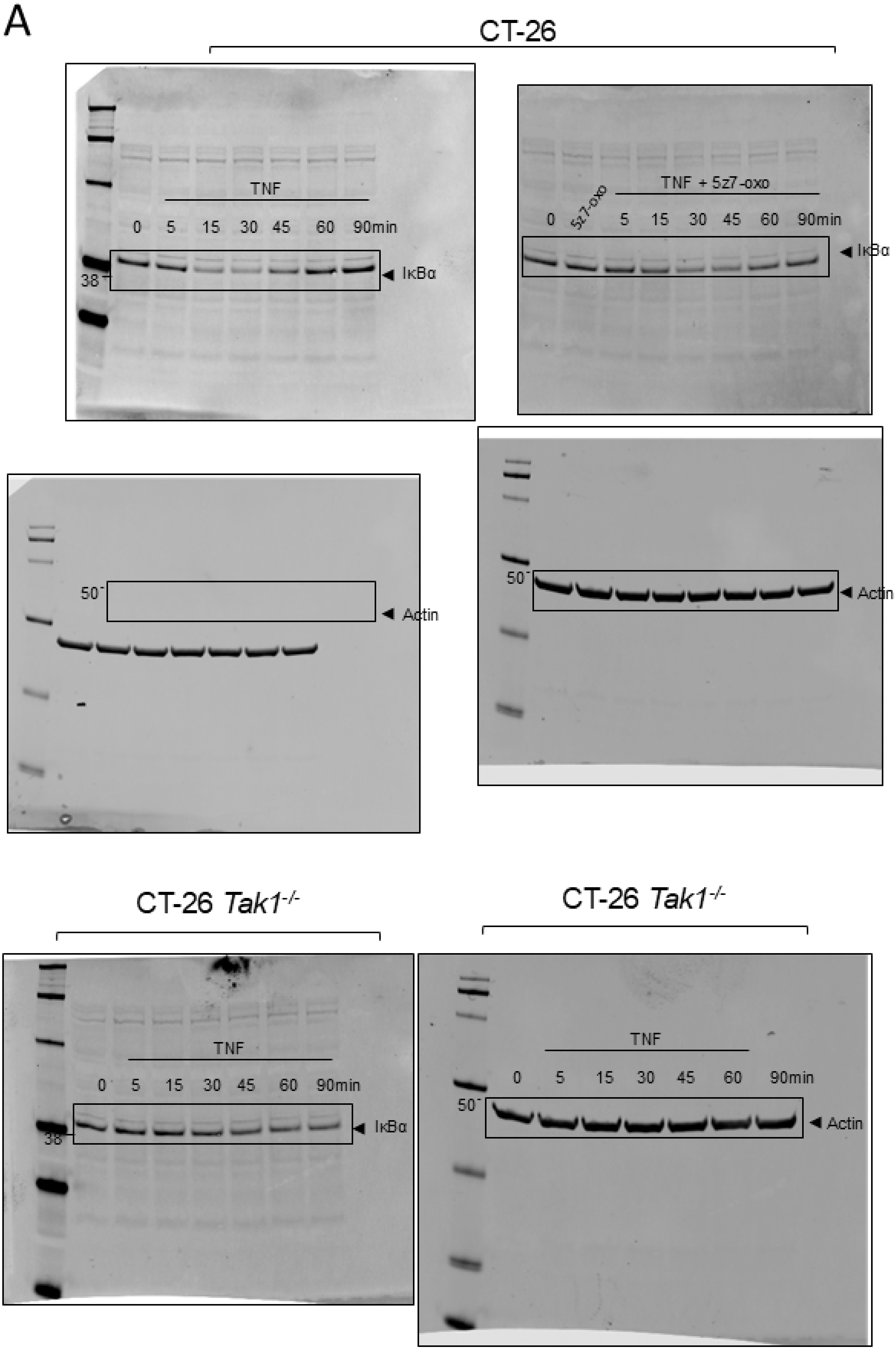

Source Data Supplementary Figure 5

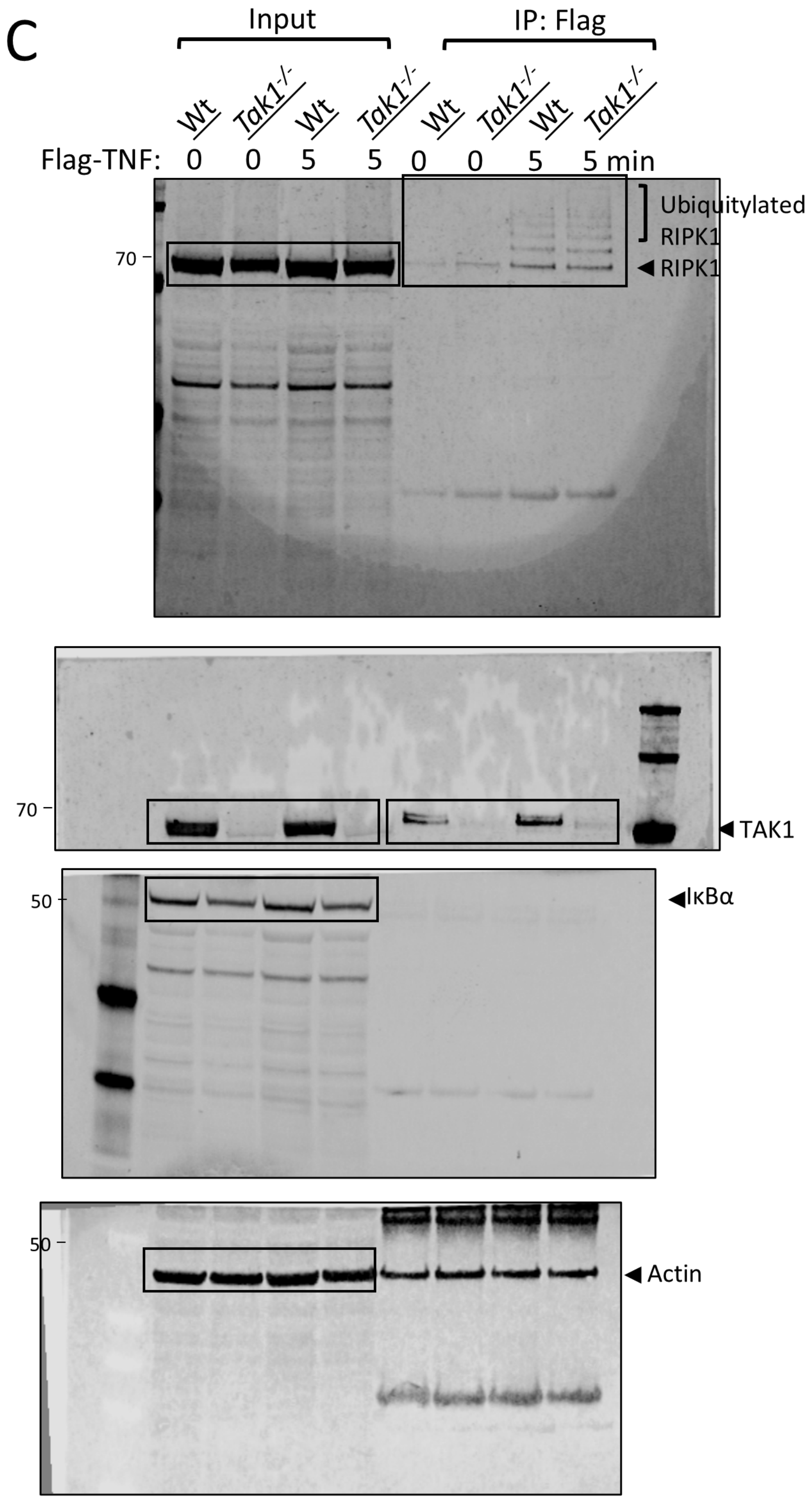

Source Data Supplementary Figure 5

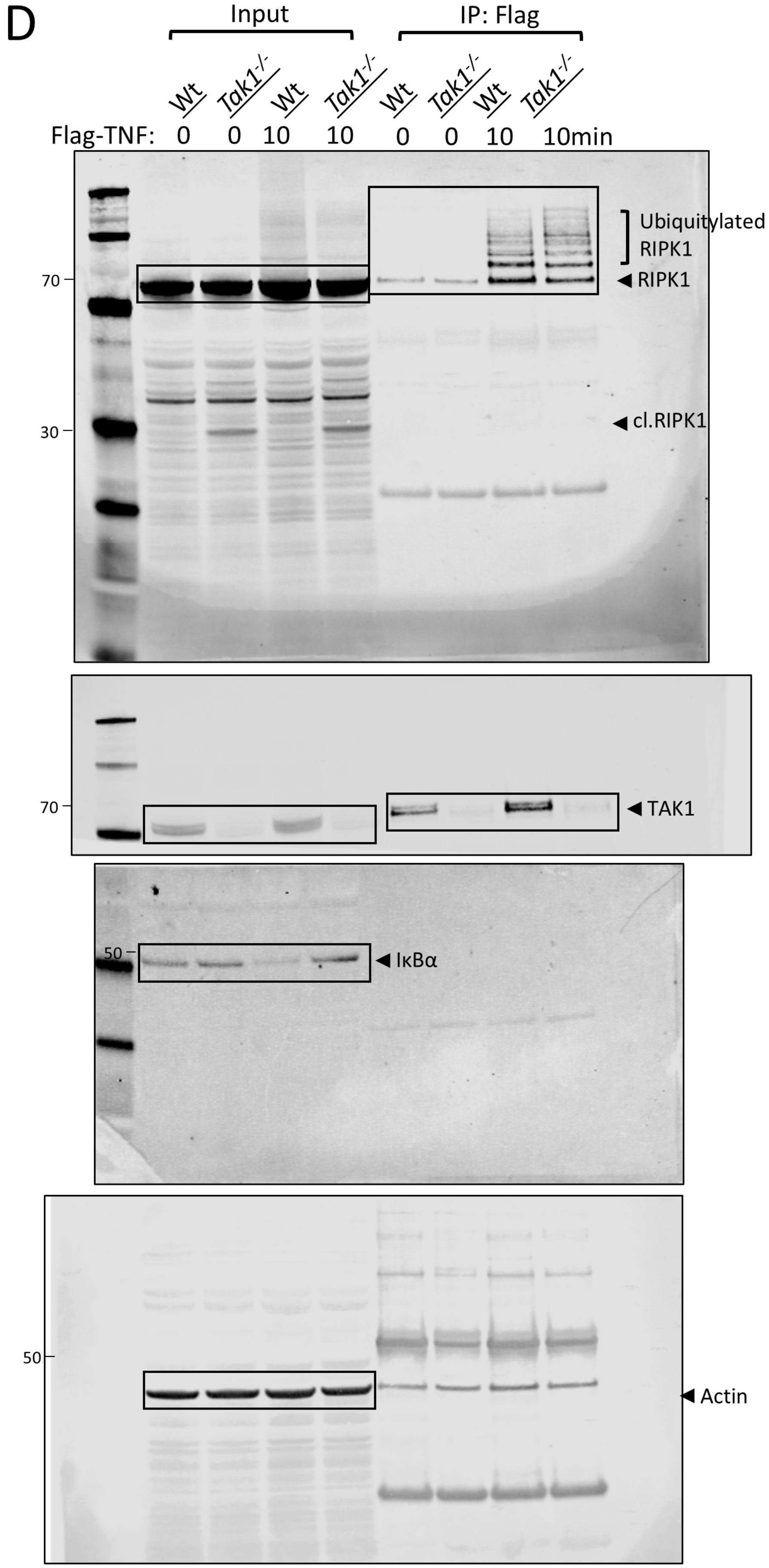

Supplement: Supplementary file 2 — Uncropped westerns [file 41419_2025_8013_MOESM2_ESM.pdf]
